# Early *in vitro* results indicate that de-O-acetylated sialic acids increase Selectin binding in cancers

**DOI:** 10.3389/fonc.2024.1443303

**Published:** 2024-12-09

**Authors:** Kakali Das, Megan Schulte, Megan Gerhart, Hala Bayoumi, Delayna Paulson, Darci M. Fink, Colin Parrish, Rachel Willand-Charnley

**Affiliations:** ^1^ Department of Chemistry, Biochemistry and Physics, South Dakota State University, Brookings, SD, United States; ^2^ Department of Microbiology and Immunology, Baker Institute for Animal Health, College of Veterinary Medicine, Cornell University, Ithaca, NY, United States

**Keywords:** de-O-acetylated sialic acid, cancer, selectins, sia-selectin pathway, Sialyl Lewis X, PSGL-1, metastasis, migration

## Abstract

Cancers utilize a simple glycan, Sialic Acid, to engage in metastatic processes via the Sialic acid (Sia) -Selectin pathway. Selectins recognize and bind to sialylated substrates, resulting in adhesion, migration, and extravasation, however, how deviations from the canonical form of Sia regulate binding to Selectin receptors (E, L, and P) on hemopoietic cells resulting in these metastatic processes, remained a gap in knowledge. De-O-acetylated Sias has been recently shown to be an integral substrate to the binding of sialic acid binding proteins. The two proteins responsible for regulating the acetyl functional group on Sia’s C6 tail, are Sialic acid acetylesterase (SIAE) and Sialic acid O acetyltransferase (CASD1). To elucidate the contribution of functional group alterations on Sia, 9-O and 7,9-O-acetylation of Sia was modulated via the use of CRISRP-Cas9 gene editing and with Sialyl Glycan Recognition Probes, to produce either O-acetylated-Sia or de-O-acetylated- Sia, respectively. *In vitro* experiments revealed that increased cell surface expression of de-O-acetylated- Sia resulted in an increase in Selectin binding, enhanced cell proliferation, and increased migration capabilities both in lung and colon cancer. These results delineate for the first time the mechanistic contribution of de-O-acetylated-Sia to Selectin binding, reinforcing the importance of elucidating functional group alterations on Sia and their contribution. Without accurate identification of which functionalized form of Sia is being utilized to bind to sialic acid binding proteins, we cannot accurately produce glycan therapeutics with the correct specificity and reactivity, thus this work contributes an integral component in the development of promising therapeutic avenues, for example in the realm of enzyme antibody drug conjugates.

## Introduction

Cancer uses simple sugar residues to engage in metastatic processes (1) via the Selectin- sialic acid (Sia) pathway, however, the mechanism of action remained a gap in knowledge (2). Although it is known that Selectins recognize and bind to sialylated substrates, resulting in adhesion, migration, and extravasation, how deviations from the canonical form of Sialic acid (Sia) regulate binding to Selectin receptors (E, L, and P) on hemopoietic cells resulting in metastatic processes, remained unexplored. It has been demonstrated in earlier research that all three Selectin work collaboratively to facilitate tumor metastasis (3). In particular, L-Selectin promotes myeloid cell recruitment and P-Selectin helps tumor cell adherence through platelet-tumor cell interactions. Together with E-Selectin, this coordinated activity promotes tumor extravasation (3, 4).

**Figure 1:**
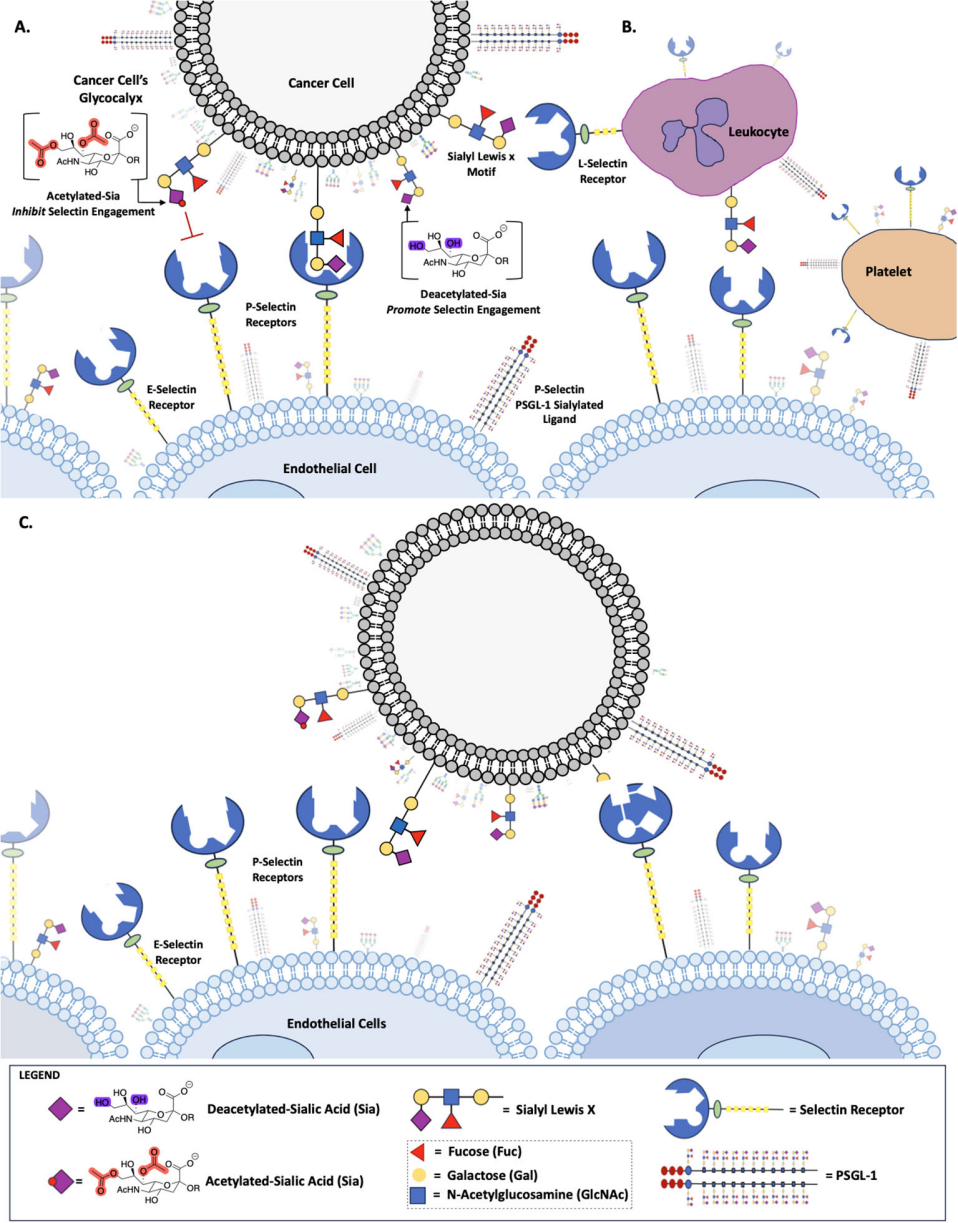
Role of Sialic Acid in Selectin Binding. Deacetylated sialic acid in the Sialyl Lewis X Motif on the cell surface promotes binding to Selectin receptors, while acetylated sialic acid inhibits this interaction **(A)**. Deacetylated Sialic acid facilitates cancer cell binding to L-Selectin on leukocytes, P-Selectin on platelets and E-Selectin on endothelial cells **(B)**. Selectin-mediated binding allows cancer cells to adhere to and migrate along the endothelium, promoting metastasis **(C)**.

Although Selectins have received a lot of attention for their role in metastasis, little is known about how non- canonical sialic acid functions, as a Selectin ligand, to modulate Selectin binding and subsequent metastasis. Metastasis, a multifaceted process that underlies the progression and lethality of cancer, involves the dissemination of cancer cells to distant sites within the body through a series of complex steps (5, 6). A significant factor underpinning cancer’s ability to engage in metastatic processes is through altered cell surface glycosylation (7–11). For instance, the glycan Sia, the specific functional groups on Sia, the enzymes that regulate those functional groups and Sia associated glycosidic linkages are adeptly utilized by cancers to engage in tumorigenic processes. Previously, for example, we showed that overexpressing de-O-acetylated-Sias on colon and lung cancers resulted in immune evasion through the inactivation of NK mediated cytotoxicity (12–14) and, separately, that deacetylated-Sia present on ABC efflux transport proteins underpinned cancer’s ability to engage in multi-drug resistance (12–14). Additionally, and of relevance to this work, the modulation of the acetyl functional group on Sias results in an increase in α-2,3-linked Sias in colon and lung cancers (12). This is of particular concern because α-2,3-linked Sias are the preferred glycosidic linkages found on Sialyl Lewis X (sLeX) and Sialyl Lewis A (sLeA) motifs, glycan substrates that bind to Selectin receptors, key glycan binding receptors identified in early metastatic processes (15–18). Motivated by our previous findings we set out to determine how de-O-acetylated- sLeX and Sialyl sLeA were being utilized by cancers to engage Selectin receptors resulting in early metastatic processes, such as migration. Our work demonstrates that de-O-acetylated-Sia bound with higher affinity to glycan binding Selectin receptors when present on the surface of colon and lung cancer cells, increasing migration (**Figure 1**).

Sialic acid, a 9-carbon keto sugar that can be highly functionalized, forms the foundation for various glycan motifs integral to cis and trans communication (12, 14, 18), like sLeX and sLeA (**Figure 1**), as well as a wide array of alterations to the sialic acid backbone itself, such as adds of: O-acetyl group on C7, C8, or C9, C5 hydroxyl groups, C5 amine modifications or C5 N-glycoyl modifications (19). Certain functional group modifications have been shown to increase the tumor’s plasticity, as demonstrated in our previous work (12, 20). Furthermore, aberrant expression of sialoglycans, has been linked to metastasis and tumor aggressiveness (8, 11, 21, 22) playing a central role in various cellular interactions, including adhesion and migration, contributing to the invasive nature of cancer cells (8, 12, 14).

Selectin receptors, calcium-dependent molecules commonly found on endothelial cells, leukocytes, and platelets, are integral to cell adhesion processes as they recognize specific glycan structures, such as fucosylated and sialylated sLeX and its isomer sLeA (23) expressed on scaffold glycoproteins serving as functional counter-receptors (24), allowing for slow tethering, and rolling of leukocytes, for instance, on the vascular endothelium (3, 25). These molecules belong to the family of Sia-binding lectin receptors and are recognized as type I membrane proteins with a C-type lectin domain (26–29)There are three types of Selectin, each specific to cell types (3, 29). P-Selectin, expressed on activated platelets and endothelial cells, has a strong preference for PSGL-1, known for its sulfated tyrosine, and a lesser affinity for sLeX motifs (30, 31). E-Selectin, found on endothelial cells, primarily binds N-glycans with sLeX motifs (31), while L-Selectin, expressed on most leukocyte types, prefers sulfated ligands, heparan-sulfate chains, and sLeX (4, 32).

All Selectin receptors recognize sLeX-bearing PSGL-1 as their ligand (3, 23) Research has shown the involvement of Selectin in the initial stages of the leukocyte- endothelial adhesion cascade, particularly in regions of inflammation (4, 11, 33). Numerous studies have also suggested the role of Selectin in metastatic processes (34, 35), indicating that cancer cells may employ a similar mechanism involving Selectin-ligand interactions for metastasis (11). This may be attributed to the fact that during the invasion stage of metastasis, where cancer cells infiltrate neighboring tissues and access blood and lymphatic vessels, Selectin-ligand interactions facilitate processes such as tethering, rolling, and signaling cascades (4).

While it is established that E, P, and L Selectin recognize sLeX and sLeA motifs on sialylated glycans, as well as how additional functional groups contribute to Selectin binding, for example, sulfated O-glycans serve as critical binding sites for Selectin. Similarly, sLeX can be modified by adding sulfate groups to the C6 position of either N-acetylglucosamine (GlcNAc) or galactose (Gal), or to both, resulting in 6-sulfo-sLeX, 6’-sulfo-sLeX or 6’, 6-bisulfo-sLeX respectively, it remains unclear how sialic acid modifications, such as O-acetylation or de-O- acetylation at the either the C9 position or C7 and C9 positions collectively, may regulate metastasis through Selectin binding.

In this study, based on prior work, we hypothesize that de-O-acetylated sialic acid engages in metastatic processes through Sia-Selectin binding metastasis via Selectin (36). To test this hypothesis, we employed CRISPR-Cas9 gene editing to create HCT116, A549, and HEK293 cell lines with specific mutations in sialic acid- related enzymes, specifically sialic acid acetyl esterase (SIAE) and sialic acid O-acetyltransferase (CASD1), both involved in the modulation of the acetyl group on sialic acid (Neu5Ac) at C7 and C9. Knocking out SIAE gene generated cancer cells with O-acetylated sialic acid, while knocking out CASD1 gene resulted in cancer cells with de-O- acetylated sialic acid. Our methodology encompassed a series of experiments, including flow cytometry for the analysis of expression levels of Selectin ligands sLeX and PSGL-1, Selectin ligand interactions, migration assays, and assessments of metabolic activity. Additionally, we investigated the impact of removing 9-O-acetyl functional groups from lung and colon cancer cell surfaces via esterase treatment on Selectin-ligand interactions.

## Materials and methods

### Scientific rigor

Selection of reagents/antibodies/cell lines for experiments were based on published literatures. The reagents were obtained from companies that provide validation. All studies include the applicable secondary antibody controls. Experiments were performed in technical triplicates with biological triplicates to confirm results. For statistical analysis we used Two-way ANOVA to analyze the data of these experiments for comparison using GraphPad Prism 8. Data, as seen below, is always presented as mean ± standard deviation with P < 0.05 indicating significance (12).

### Cell lines and cell culture

All cell lines were purchased from American Type Culture Collection. A549 and HEK 293 cells were grown in Dulbecco modified Eagle medium (DMEM Corning) with 10% fetal bovine serum (Corning) and 1% pen/strep (Cytiva Hyclone). HCT 116 cells were grown in RPMI 1640 medium (Corning) with 10% fetal bovine serum and 1% pen/strep. Concerning preparing cell suspension for assays, Cell Dissociation Buffer (Gibco, Waltham, MA) was used for dissociation of cells as it does not disrupt the glycocalyx. Cell culture was not permitted to exceed 80% confluency in the flask to avoid genetic drift. The CASD1 (ΔCASD1) and SIAE knockout (ΔSIAE) A549 cell lines were obtained from Cornell University and used as previously reported. The CASD1 (ΔCASD1) and SIAE knockout (ΔSIAE) HCT116 cell lines were used as previously reported (12, 14). Cells are routinely subject to authentication (using a variety of methods and are not permitted to be utilized for experimentation beyond 20 passages). Additionally, all cell lines are tested for mycoplasma (ATCC 30-1012K) bi-weekly according to published protocol. Additionally, Mycoplasma testing occurs prior to entering cryo and prior to beginning experimentation. Aseptic tissue culture training and techniques are strictly adhered to, to diligently avoid contamination.

### Cell surface ligand staining assay

Cells are grown to 80% confluency, upon which time media is removed, and cells are washed three times gently with PBS prior to treatment with Cell Dissociation Buffer (Gibco, Waltham, MA). Cells are then collected, centrifuged, counted, resuspended in Cell Staining Buffer (Bio Legend) and plated in a 96 well V-bottom plate at 1x10^5^ cells per well and subject to further treatment. 5μL of Human TruStain FcX™ (Fc Receptor Blocking Solution, Bio Legend) is added per 100μl of cell suspension and incubated for 30 minutes at room temperature. Cells are spun down to remove excess solution and resuspended in dilutions between 0.1-10 μg/ml of FITC anti-human Sialyl Lewis X (dimeric) Antibody (Bio Legend) or FITC anti-PSGL-1 antibody [TC2] (abcam). Dilutions were made in FACS buffer (1XPBS, 0.5% BSA, 1mM CaCl2). Cells are then incubated at 4°C for 1 hour (sLeX) or 2 hours (PSGL-1). The cells are then washed twice with 200μL of Cell Staining Buffer (Cat. No.420201, BioLegend), resuspension and treated with 7-AAD Viability Staining Solution (Bio Legend) prior to analyzed via flow cytometry. All flow cytometry data was analyzed using FlowJo v. 10.0 (Tree Star, Ashland, OR). All experiments were performed in three technical and biological replicates to ensure reproducibility.

### Preparation of Selectin-FITC antibody conjugates

Selectin-FITC antibody conjugates were prepared at a concentration of 30µg/mL of Selectin per 5x10^5^ cells in 1X FACS buffer (1X PBS supplemented with 0.5% BSA and 1mM CaCl2). Secondary Fc conjugate (abcam) was added at an equal concentration to Selectin (R&D Systems) resulting in the final suspension containing 30 µg/mL concentration of Selectin-FITC antibody conjugates in FACS Buffer.

### Selectin binding assay

Cells were grown to 80% confluency then washed gently three times with PBS prior to treatment with Cell Dissociation Buffer (Gibco, Waltham, MA). Cells were centrifuged, counted, and resuspended in appropriate media at concentration of 1x10^6^ cells/mL. Cells were plated into a 96 well V-bottom plate at 2x10^5^ cells per well. Cells were centrifuged at 200 x g for 5 minutes. Media was removed and cells were washed two-three times with 100μL of FACS Buffer. Next, cells were treated with 50μL of Selectin-Fc conjugate. Cells were incubated in the dark at 4°C for one hour. After the incubation, the cells were centrifuged and washed once with 100μL of FACS buffer. The final resuspension was in 200μL of FACS Buffer. Cells were analyzed via flow cytometry. All flow cytometry data was analyzed using FlowJo v. 10.0 (Tree Star, Ashland, OR). All experiments were performed in three technical and biological replicates (4).

### 7,9-deacetylated-Sia esterase probe assay

The control cell lines, of A549, HCT116, and HEK 293, were grown to 80% confluency and washed gently three times with PBS and treated with Cell Dissociation Buffer (Gibco, Waltham, MA). Cells were then centrifuged, counted, and resuspended in appropriate media at a concentration of 1x10^6^ cells/mL. Cells were plated into a 96 well V-bottom plate at 2x10^5^ cells per well, and centrifuged at 200 x g for 5 minutes. Media was then removed, and cells were washed twice with 100μL of FACS Buffer and resuspended in same buffer. For esterase treatment, cells were incubated with the requisite Sialyl Glycan Recognition probes. Specifically, virolectin esterases that selectively bind and remove 9-O-acetyl Sia (porcine torovirus, PTOV) and 7,9-O-acetyl Sia (bovine coronavirus, BCoV), at 37°C with 5% CO2 for 90 minutes. Cells were then centrifuged, the probe was removed, and the cells were washed prior to treatment with Selectin-FITC conjugates (12, 14).

### Scratch migration assay

Cells grown to 80% confluency were washed once with PBS and treated with Cell Dissociation Buffer (Gibco, Waltham, MA). Cells were centrifuged, counted, and resuspended in appropriate media. 7.5x10^4^ cells were plated into an ibidi insert with a 500 µm divider. Cells were grown until confluent (overnight) and the divider was removed. Cells were imaged using an EVOS XL Core imaging system immediately after removing the insert (0 hours) and at 12, 24, 36, and 48 hours, respectively.

### Proliferation assay

Cells were grown to 80% confluency, upon which time the media was removed, the cells were washed once with PBS and treated with Cell Dissociation Buffer (Gibco, Waltham, MA). Cells were centrifuged, counted, and resuspended in appropriate media. Control cell lines, ΔSIAE, and ΔCASD1 were plated at 5x10^4^ cells in a 12 well plate with 0.5 mL of media containing 10% Fetal Bovine Serum (FBS). Cells were incubated at 37°C and 5% CO2 for two hours in the dark. Next, 50μL of MTT and 50μL media were added to each well and incubated at 37°C and 5% CO2 for 2 hours. 150μL of MTT solvent was added to each well and rocked for 15 min in the dark. Absorbance was measured at 590nm using a microplate reader (Cytation, BioTek).

### Transwell migration assay

Transwell migration stimulated with 0.2% FBS-DMEM (negative control), 10% FBS-DMEM (positive control), L-Selectin supplemented DMEM (10% FBS-DMEM), or P-Selectin supplemented DMEM (10% FBS-DMEM) was quantified in lung cancer cell lines (A549 Control, A549 ΔSIAE, A549 ΔCASD1) as well as colon cancer cell lines (HCT116 Control, HCT116 ΔSIAE, HCT116 ΔCASD1). 40,000 cells suspended in 100µL 0.2% FBS-DMEM were seeded in the upper chamber of Transwell permeable supports within a 24-well plate (Corning, Cat No 3464) onto a pre-soaked, equilibrated PET membrane with 8 μm pores. 500µL of stimulus containing or control media was placed in the lower chamber. After 24hr incubation at 37˚C and 5% CO2, non-migratory cells were removed from the top of the membrane using a cotton swab. The membrane was then rinsed once with 1X PBS and fixed with methanol. After drying completely, membranes were mounted in Fluoromount G with DAPI (SouthernBiotech, Cat no. 0100-20) and imaged via epifluorescence microscopy using a Nikon Eclipse E800 epifluorescence microscope and a Zeiss Axiocam 503 monochrome camera. Three images were acquired per membrane for a total of approximately 100 quantified cells per technical replicate. DAPI labeled nuclei of migratory cells were quantified using CellProfiler’s “Identify Primary Objects” function, and statistical analysis was completed using GraphPad Prism. Quantifications for these assays are from one replicate that is representative of three biological replicates, where each data point represents one quantified membrane. Each biological replicate consisted of three technical replicates (three membranes) per stimulus for each cell line. A549 ΔSIAE, A549 ΔCASD1, HCT116 ΔSIAE, and HCT116 ΔCASD1 migration are graphed relative to 10% FBS-DMEM stimulated A549 Control and HCT116 Control migration, respectively.

## Results

### Increased expression of Selectin ligand PSGL-1 on cell surface of HCT116 and A549 cancer cell lines in the presence of deacetylated sialic acid

**Figure 2:**
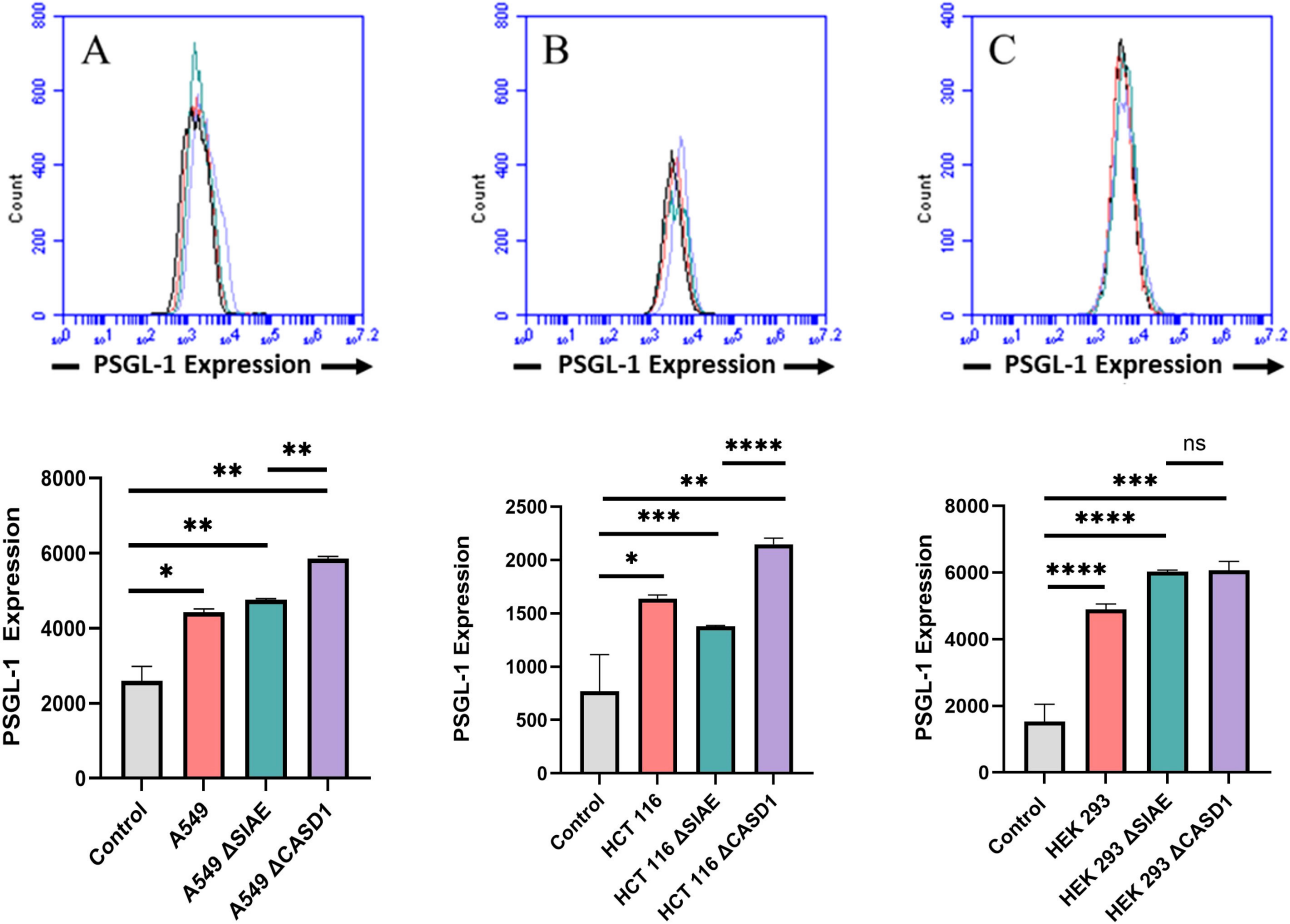
Expression Levels of P-Selectin Glycoprotein Ligand-1 (PSGL-1) on the Surface of Lung, Colon, and Control Lines. PSGL-1 expression was assessed in A549 lung cancer cells **(A)**, HCT 116 colon cancer cells **(B)**, and HEK 293 non-cancerous kidney cells **(C)**, along with their respective knockouts. Cells were plated in a 96-well V-bottom plate and treated with Tru-Stain blocking buffer for 30 minutes. Subsequently, cells were incubated with commercially available PSGL-1-FITC for 2 hours at 4°C in the dark. Data were collected via flow cytometry. For statistical analysis was conducted using Two-way ANOVA to analyze the data of these experiments for comparison using GraphPad Prism 8. Data, as seen below, is always presented as mean ± standard deviation with P < 0.05 indicating significance. Error bars represent the standard deviation of triplicate samples. * p<0.05, ** p<0.005, *** p<0.001, ****p<0.0001, ns = not significant (p>0.05).

P-Selectin glycoprotein ligand-1 (PSGL-1) is a dimeric mucin recognized by its distinctive sulfated tyrosine structure (37, 38). PSGL-1 is a common ligand for all three E, P and L Selectin and has been shown to promote metastasis by binding to Selectin (4, 39). In this study, we set out to investigate the expression of PSGL-1 in A549, HCT116 and HEK-293 cell lines, along with knockouts of SIAE and CASD1. Our findings revealed the highest cell surface expression of PSGL-1 in the HCT116 ΔCASD1 and A549 ΔCASD1 cell lines (**Figure 2**).

### Increased expression of Selectin ligand, sialyl Lewis X, on cell surface of HCT116 and A549 cancerous cell lines in the presence of de-O-acetylated sialic acid sialic acid

**Figure 3:**
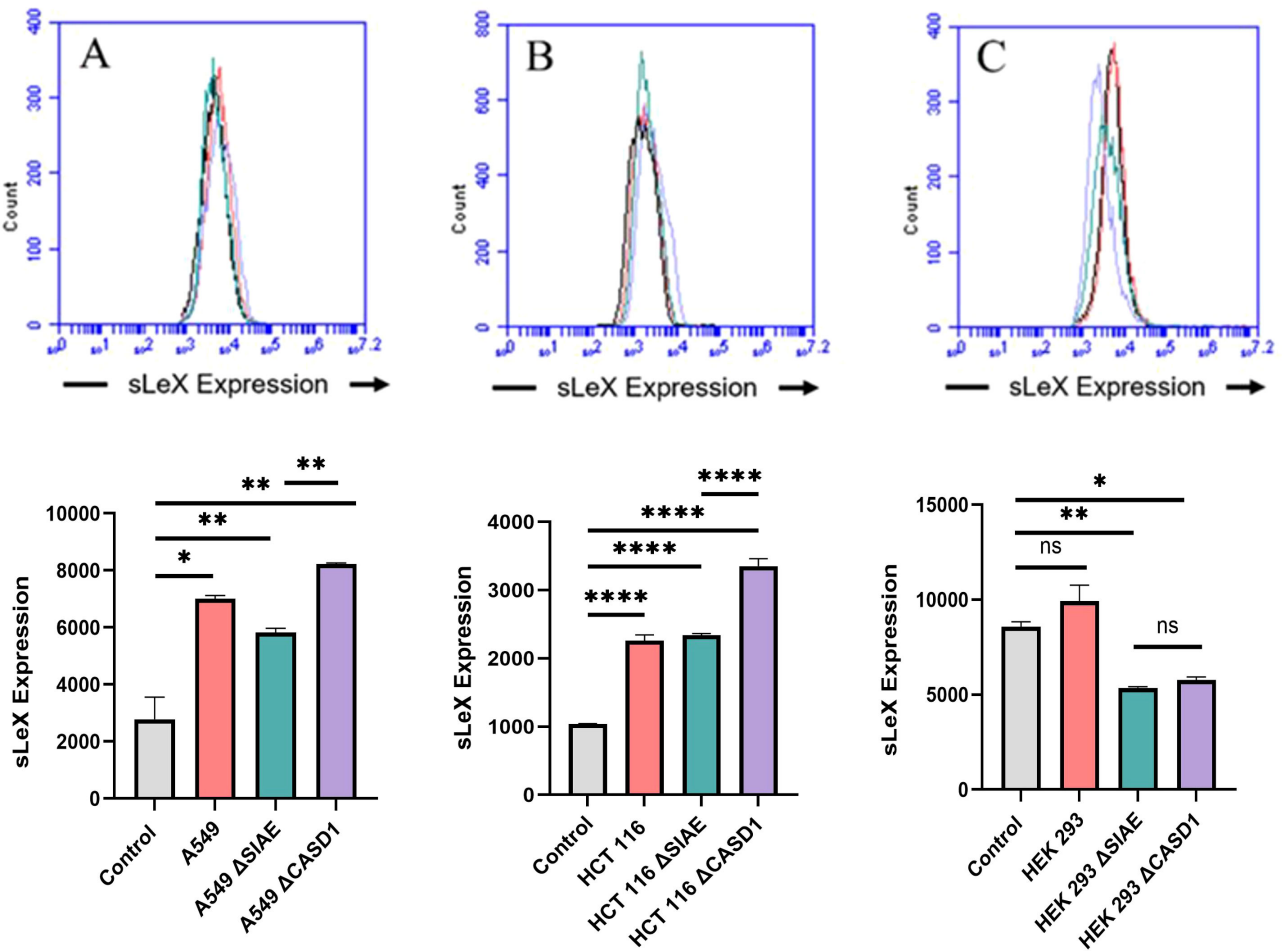
Expression Levels of Sialyl Lewis X (sLeX) on Lung, Colon, and Control Lines. sLeX expression was assessed in A549 lung cancer cells **(A)**, HCT 116 colon cancer cells **(B)**, and HEK 293 non-cancerous kidney cells **(C)**, along with their respective knockouts. Cells were plated in 96-well V-bottom plates and treated with Tru-Stain blocking buffer. Cells were then treated with commercially available sLeX-FITC and incubated for 1 hour at 4°C in the dark. Data was collected via flow cytometry. For statistical analysis was conducted using Two-way ANOVA to analyze the data of these experiments for comparison using GraphPad Prism 8. Data, as seen below, is always presented as mean ± standard deviation with P < 0.05 indicating significance. Error bars represent the standard deviation of triplicate samples. * p<0.05, ** p<0.005, ****p<0.0001, ns = not significant (p>0.05).

Selectin receptors are known to bind with sialic acid containing motifs, with sLeX being a prominent glycan motif underpinning Sia-Selectin interaction and binding. Prior to cell surface selectin binding assays, we needed to determine the presence of sLeX motifs. Thus, we determined set out to determine the cell surface expression of sLeX in A549, HCT116 and HEK-293 cell lines, along with knockouts of SIAE and CASD1. Our findings revealed that sLeX is present on the cell’s surface with highest expression being on the HCT116 ΔCASD1 and A549 ΔCASD1 cell lines (**Figure 3**). The presence of sLeX on A549 line aligns with previous findings (40). Additionally, sLeX has been previously detected in colon and hepatic carcinoma cells, particularly on core 2 O-glycans (41).

### De-O-acetylation of sialic acid increases E-, P-, and L-Selectin binding of lung and colon cancer cell lines

**Figure 4:**
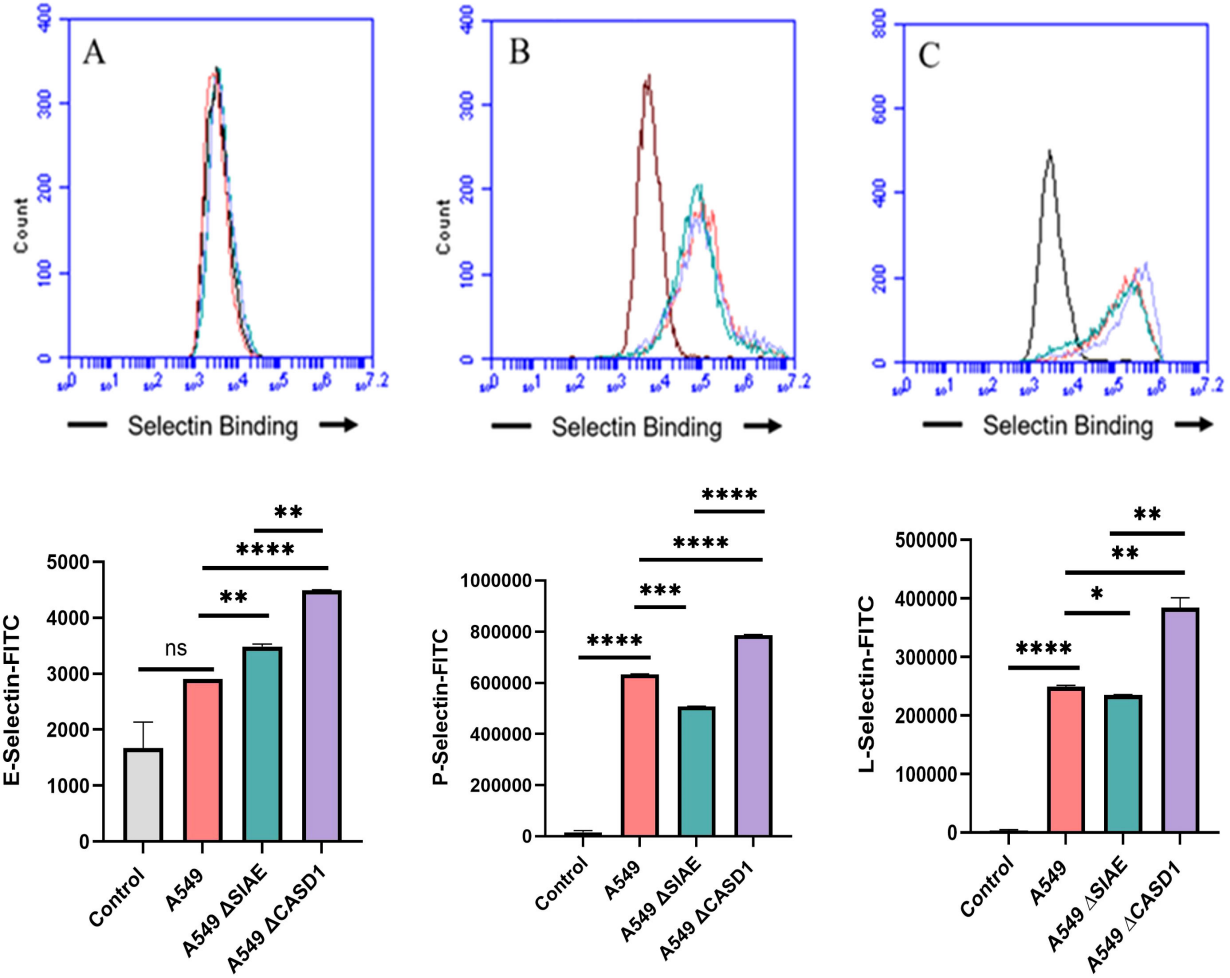
Selectin Binding Assay on Lung Cancer. Binding interactions between E-Selectins **(A)**, P-Selectins **(B)**, and L-Selectins **(C)** with their respective ligands were quantified using flow cytometry. A549 cells were seeded at a density of 2x10^5 cells per well in a 96-well V-bottom plate and incubated with different Selectins-FITC conjugates at 4°C for one hour. For statistical analysis was conducted using Two-way ANOVA to analyze the data of these experiments for comparison using GraphPad Prism 8. Data, as seen below, is always presented as mean ± standard deviation with P < 0.05 indicating significance. Error bars represent the standard deviation of triplicate samples. * p<0.05, ** p<0.005, *** p<0.001, ****p<0.0001, ns, not significant (p>0.05).

**Figure 5:**
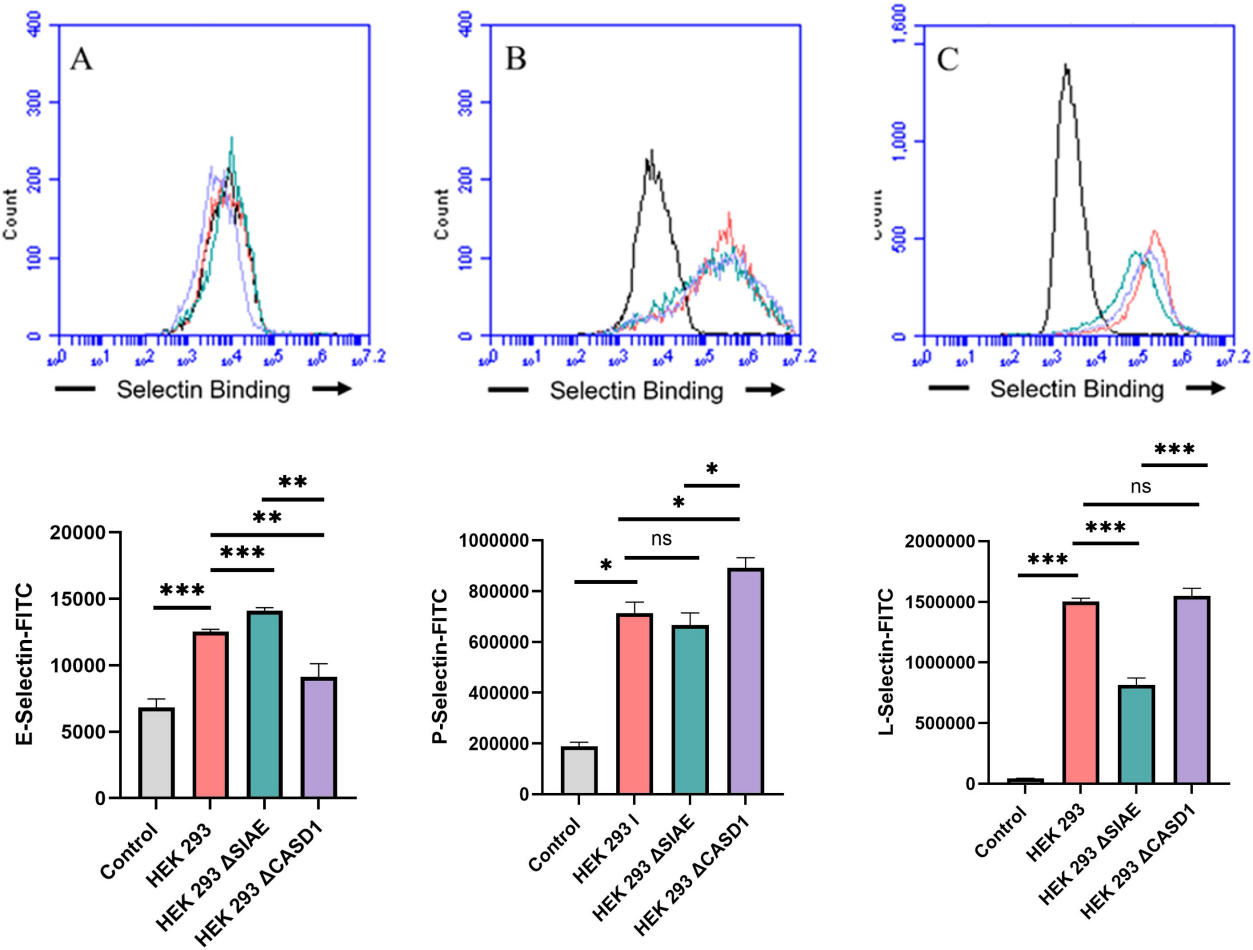
Selectin Binding Assay on Non-Cancerous Kidney Cells. Binding interactions between E-Selectins **(A)**, P-Selectins **(B)**, and L-Selectin **(C)** with their respective ligands were quantified using flow cytometry. HEK 293 cells were seeded at a density of 2x10^5 cells per well in a 96-well V-bottom plate and incubated with different Selectins-FITC conjugates at 4°C for one hour. For statistical analysis was conducted using Two-way ANOVA to analyze the data of these experiments for comparison using GraphPad Prism 8. Data, as seen below, is always presented as mean ± standard deviation with P < 0.05 indicating significance. Error bars represent the standard deviation of triplicate samples. * p<0.05, ** p<0.005, *** p<0.001, ****p<0.0001, ns = not significant (p>0.05).

**Figure 6:**
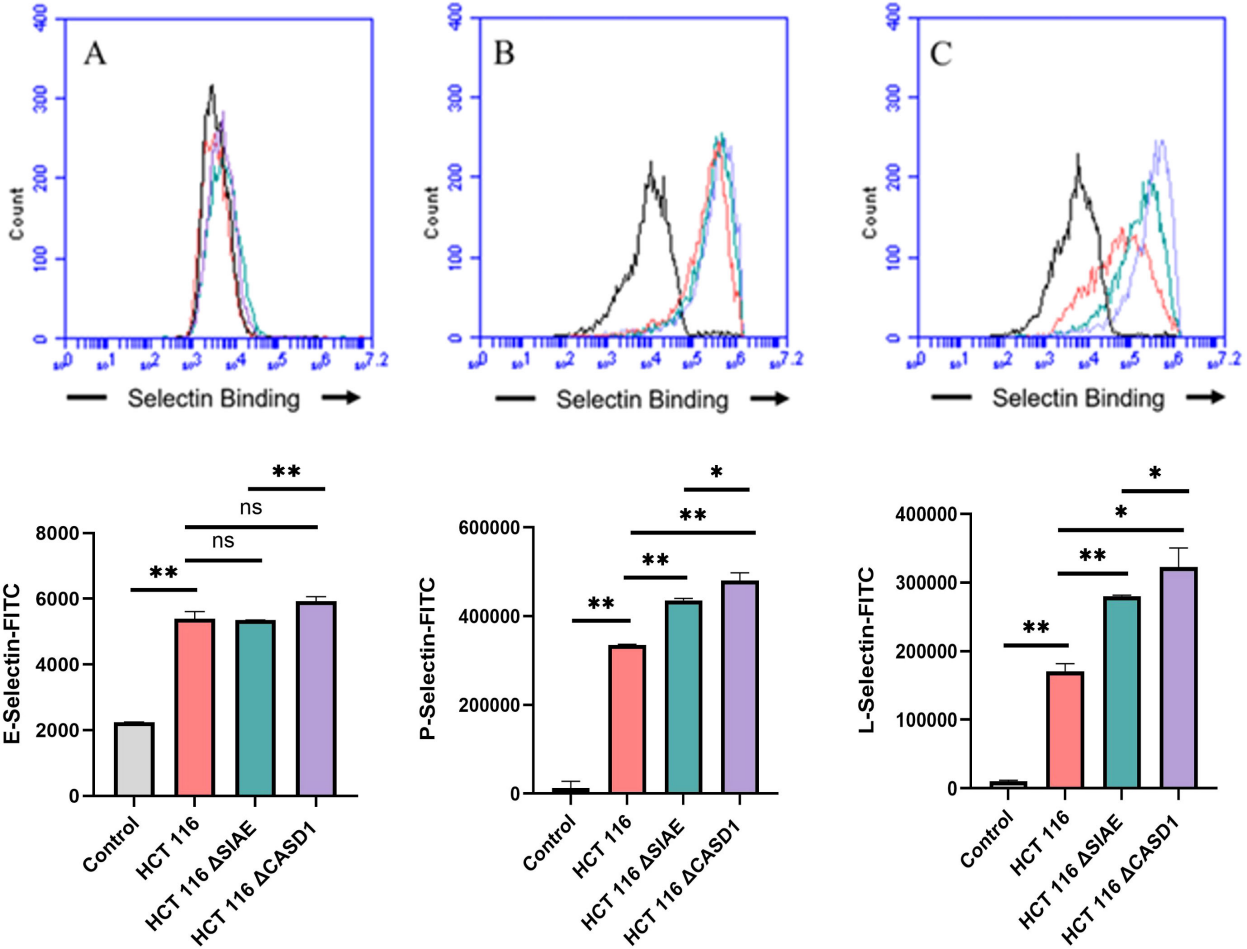
Selectin Binding Assay on Colon Cancer. Binding interactions between E-Selectins **(A)**, P-Selectins **(B)**, and L-Selectins **(C)** with their respective ligands were quantified using flow cytometry. HCT116 cells were seeded at a density of 2x10^5 cells per well in a 96-well V-bottom plate and incubated with different Selectins-FITC conjugates at 4°C for one hour. For statistical analysis was conducted using Two-way ANOVA to analyze the data of these experiments for comparison using GraphPad Prism 8. Data, as seen below, is always presented as mean ± standard deviation with P < 0.05 indicating significance. Error bars represent the standard deviation of triplicate samples. * p<0.05, ** p<0.005, *** p<0.001, ns, not significant (p>0.05).

**Figure 7:**
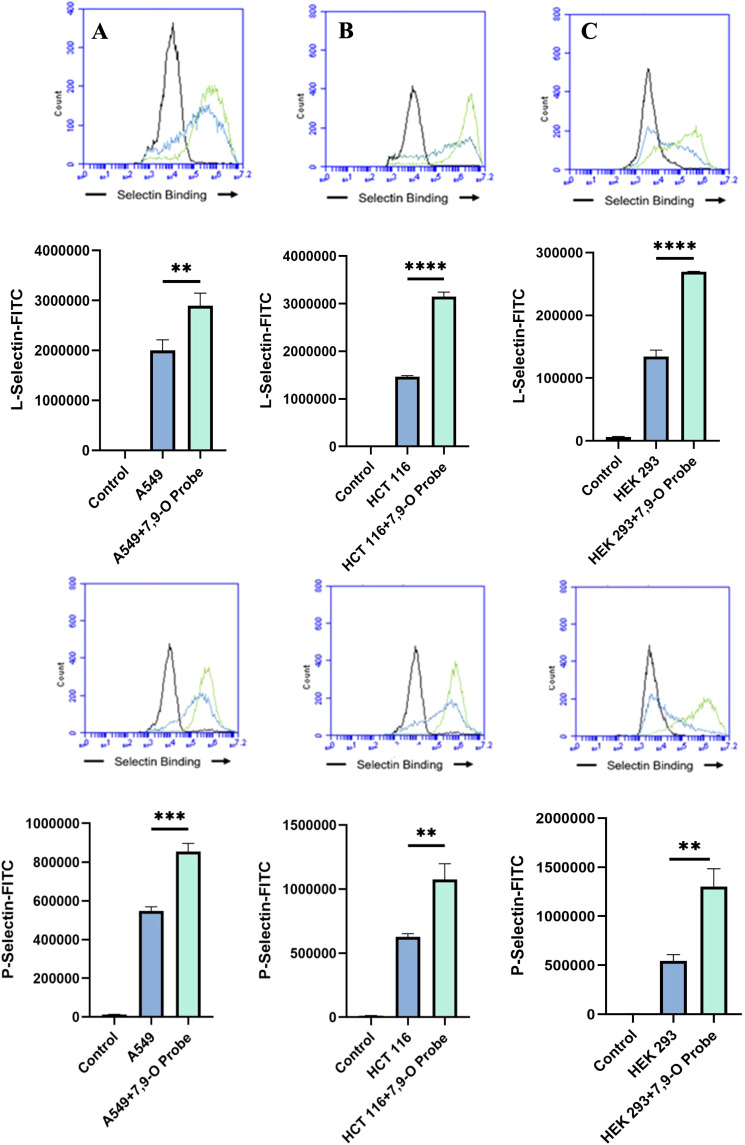
Determining the Modulation of Deacetylated-Sialic Acid to Selectin Binding via Sialylglycan Recognition Probes (SGRP). The binding of L-Selectins **(A-C)** and P-Selectins **(D-F)** to control cell lines A549, HCT 116, and HEK 293, respectively, were evaluated following treatment with an esterase that specifically removes sialic acid acetyl groups. Cells were seeded in 96-well V-bottom plates, washed with FACS buffer, and treated with 9-O-Ac probe (PTOV He-Fc, #1554). After a 1.5-hour incubation at 37°C, cells were washed again and incubated with Selectins-FITC conjugates at 4°C for 1 hour in the dark. Data were acquired using flow cytometry. For statistical analysis was conducted using Two-way ANOVA to analyze the data of these experiments for comparison using GraphPad Prism 8. Data, as seen below, is always presented as mean ± standard deviation with P < 0.05 indicating significance. Error bars represent the standard deviation of triplicate samples. ** p<0.005, ****p<0.0001, ns = not significant (p>0.05).

Upon confirming the presence of Selectin ligands on the surface of our cells, we next determined the modulatory effects of de-O-acetylated Sia has on Selectin binding. Our study aimed to investigate how the manipulation of sialic acid functional group at C7 and C9 influences Selectin receptor binding in cancer cells. As mentioned earlier, E-Selectin favors binding to sLeX motifs (31), while P- and L-Selectin are capable of binding sLeX or sLeA (48). Recent *in vivo* studies in mice have shown that metastasis of breast cancer cell line to lung does not primarily utilize E-Selectin for direct ligand binding meditated metastasis, in contrast to metastasis to bone which relies on E-Selectin ligand binding (4, 34). Our findings align with this observation, as we detected minimal ligand binding of E-Selectin in A549 lung cancer cells (**Figure 4**). However, we also observed a similar low E-Selectin binding pattern in noncancerous HEK-293 cells and HCT116 colon cancer cells (**Figures 5**, **6**). Importantly, our primary finding demonstrates that the de-O-acetylated Sia leads to an increase in Selectin binding. This effect extends to E-, P-, and L-Selectin, although it’s noteworthy that P- and L-Selectin appear to contribute the most to the phenomenon in both lung and colon cancer (**Figures 4**, **6**). To substantiate our results regarding increased Selectin binding via de-O-acetylated Sia expressing cell lines (**Figures 5**–**6**), we once again utilized our Sialyl Glycan Recognition Probes (virolectin esterase) to selectively bind and remove 9-O-acetyl Sia (porcine torovirus, PTOV) and 7,9-O-acetyl Sia (bovine coronavirus, BCoV) on control cell lines to mimic the CASD1 knockout producing de-O-acetylated Sias on the cell surface. The results substantiate previous findings that when de-O-acetylated Sia is present on the cell’s surface it significantly increases P- and L-Selectin binding in A549 and HCT116 cell lines (**Figure 7**).

### De-O-acetylation of sialic acid do not significantly increase cell proliferation

**Figure 8:**
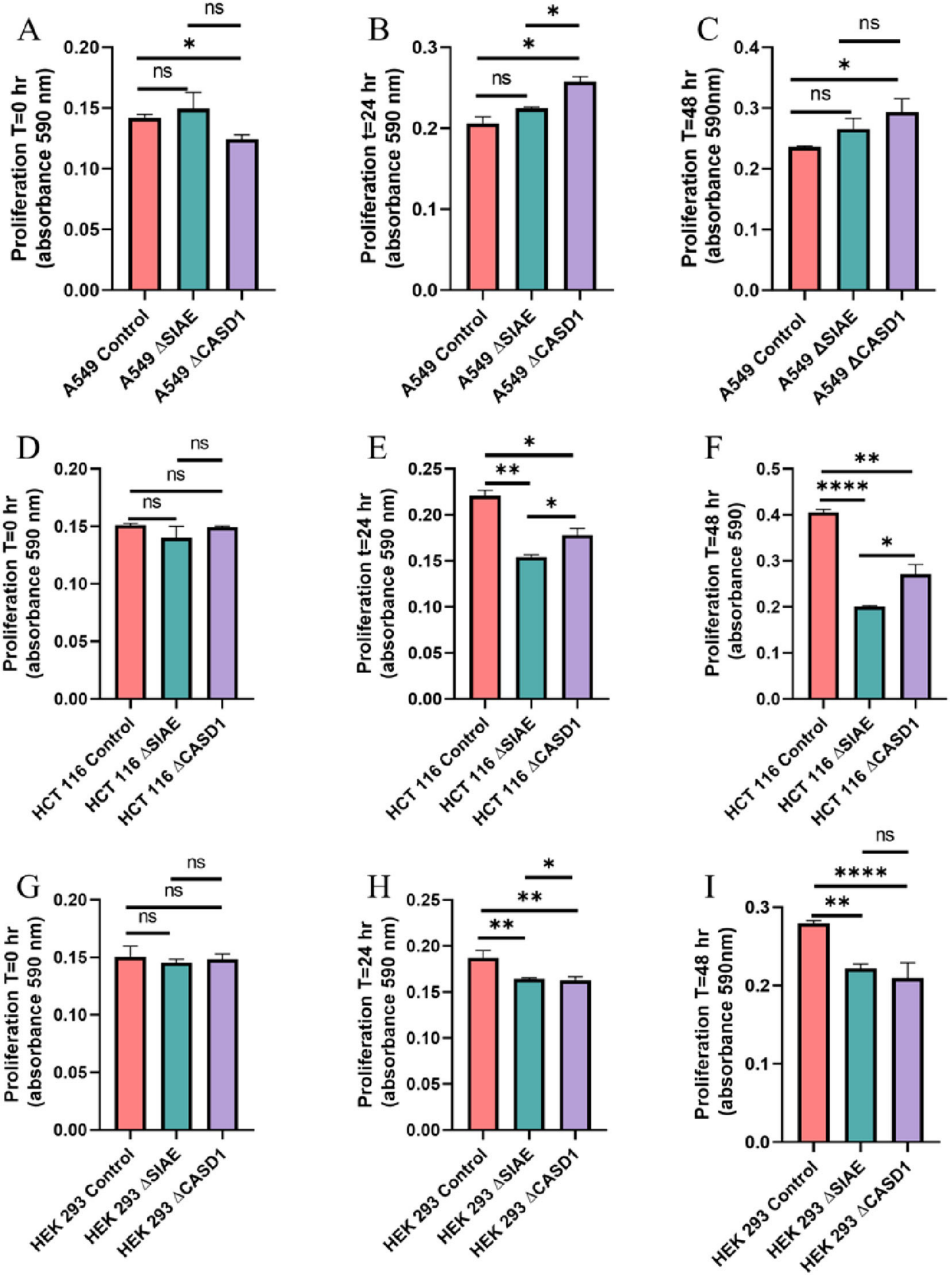
Proliferation Assay. A549 cells **(A-C)**, HCT 116 cells **(D-F)**, and HEK 293 cells **(G-I)** were plated at a density of 50,000 cells per well in separate 12-well plates containing media supplemented with 10% FBS. Cells were incubated for 0 hours (left), 24 hours (middle), and 48 hours (right). At each time point, 50 μl of MTT reagent and 50 μl of media were added to each well. After a 2-hour incubation at 37°C in the dark, 150 μl of neutralizing solvent was added, followed by rocking for 15 minutes. Absorbance was measured at 590 nm to assess cell proliferation, with increased absorbance indicating higher metabolite levels. Data were acquired using flow cytometry. For statistical analysis was conducted using Two-way ANOVA to analyze the data of these experiments for comparison using GraphPad Prism 8. Data, as seen below, is always presented as mean ± standard deviation with P < 0.05 indicating significance. Error bars represent the standard deviation of triplicate samples. * p<0.05, ** p<0.005, ****p<0.0001, ns = not significant (p>0.05).

To assess the impact modulating acetylated-Sias has cell proliferation rate, we conducted an MTT proliferation assay. No discernable effect consistently presented itself through experimentation. In lung cancer cell line A549 that modulating acetylation, whether it was de-O-acetylated-Sia (ΔCASD1) or O-acetylated-Sia (ΔSIAE) resulting in the cell proliferation being slightly increased after 24 and 48 hrs. compared to the Control cell line (**Figure 8**). In HCT116 colon cancer cells the cell proliferation was significantly lower in both ΔCASD1 and ΔSIAE compared to control after 24 and 48 hrs.(**Figure 8**). In non-cancerous HEK-293 cells a similar effect was observed as in HCT116. Cell proliferation was significantly lower in both ΔCASD1 and ΔSIAE cell lines compared to control HEK-293 (**Figure 8**). HEK-293 control cells showed the highest proliferation after 48 hrs. among three above mentioned cell lines (A549, HCT116 and HEK-293), with the SIAE and CASD1 knockout having significantly decreased proliferation relative to the control in HCT116 and HEK-293. Our data strongly suggests that cancer cells utilize sialic acid to modulate their rate of division.

### Deacetylation of sialic acid enhances the migration capabilities of lung and colon cancer cells

**Figure 9:**
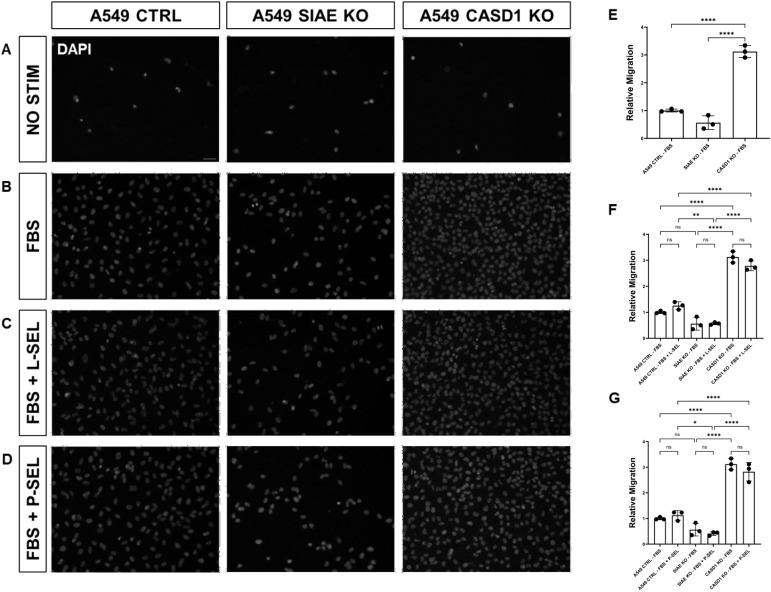
SIAE and CASD1 Positively and Negatively Regulate Cell Migration, Respectively, in A549 cells. **(A-D)** Epifluorescent micrographs of transwell membranes with DAPI labeled nuclei in A549 Ctrl, SIAE KO, and CASD1 KO cel lines. A549 lung cancer cells were seeded on a PET membrane and stimulated with 0.2% FBS-DMEM **(A)**, 10% FBS- DMEM **(B)**, L-Selectins supplemented 10% FBS-DMEM **(C)**, or P-Selectins supplemented 10% FBS-DMEM **(D)**. After 24hrs, migratory cells on the membranes’ bottom surface were fixed in methanol and mounted in media containing DAPI. Membranes were imaged via epifluorescent microscopy, and migratory cell nuclei were quantified. **(E-G)** Cell migration of SIAE and CASD1 knockout cell lines stimulated with FBS **(E)**, L-Selectins and FBS **(F)** or P-Selectins and FBS **(G)** is graphed relative to A549 Ctrl migration when stimulated with 10% FBS-DMEM. Scale bar = 100µm. Quantifications show one of three biological replicates, each including three membranes per stimulus for each cell line. *p< 0.05 indicating significance. Error bars represent the standard deviation of triplicate samples. * p<0.05, ** p<0.005, ****p<0.0001, ns = not significant (p>0.05).

**Figure 10:**
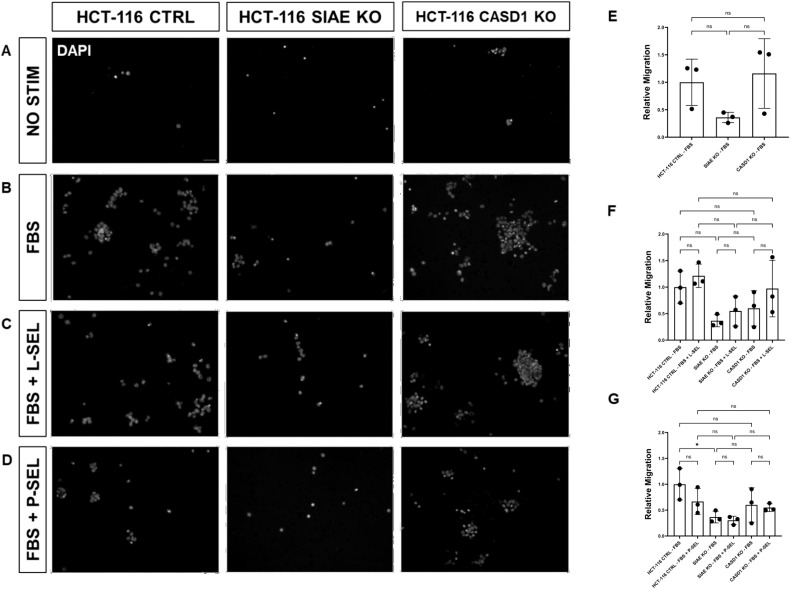
SIAE Facilitates Cell Migration in HCT-116 Cells. **(A-D)** Epifluorescent micrographs of transwell membranes with DAPI labeled nuclei in HCT-116 Ctrl, SIAE KO, and CASD1 KO cell lines. HCT-116 colon cancer cells were seeded on a PET membrane and stimulated with 0.2% FBS-DMEM **(A)**, 10% FBS-DMEM **(B)**, L-Selectins supplemented 10% FBS- DMEM **(C)**, or P-Selectins supplemented 10% FBSDMEM **(D)**. After 24hrs, migratory cells on the membranes’ bottom surface were fixed in methanol and mounted in media containing DAPI. Membranes were imaged via epifluorescent microscopy, and migratory cell nuclei were quantified. **(E-G)** Cell migration of SIAE and CASD1 knockout cell lines stimulated with FBS **(E)**, L-Selectins and FBS **(F)** or P-Selectins and FBS **(G)** is graphed relative to HCT-116 Ctrl migration when stimulated with 10% FBS-DMEM. Scale bar = 100µm. Quantifications show one of three biological replicates, each including three membranes per stimulus for each cell line. * p<0.05 ns = not significant (p>0.05) indicating significance. Error bars represent the standard deviation of triplicate samples.

We used a transwell migration assay to determine how modulation of acetylated sialic acids affected the ability of cells to migrate through a membrane bearing small pores in response to a chemoattractant placed in the lower well of a migration chamber. Upon stimulation with FBS, deacetylation of sialic acid (ΔCASD1) caused a significant increase in cell migration in A549 lung cancer cells (**Figure 9E**). In contrast, cells displaying acetylated sialic acid (ΔSIAE) showed a trend to reduced migration as compared to control A549 lung cancer cells. Similarly, in HCT116, migration of cells displaying deacetylated sialic acid (ΔCASD1) trended up, while migration trended down in the presence of acetylated sialic acid (ΔSIAE) (**Figure 10E**).The addition of soluble L- or P-Selectin to the FBS-containing migratory stimulus media in the lower well of the transwell chamber did not significantly change migration of any of the cell lines relative to FBS alone (**Figures 9F, G**, **10F, G**). This suggests that soluble Selectins are not a stimulus for chemoattraction, as would be expected for an adhesion-based migration receptor-ligand interaction such as that between sialic acid and Selectins. These findings underscore the crucial role of de-O-acetylated sialic acid in influencing cell migration.

### In the absence of Selectin binding, de-O-acetylation of sialic acid reduced the migration ability of lung and colon cancer cells

**Figure 11:**
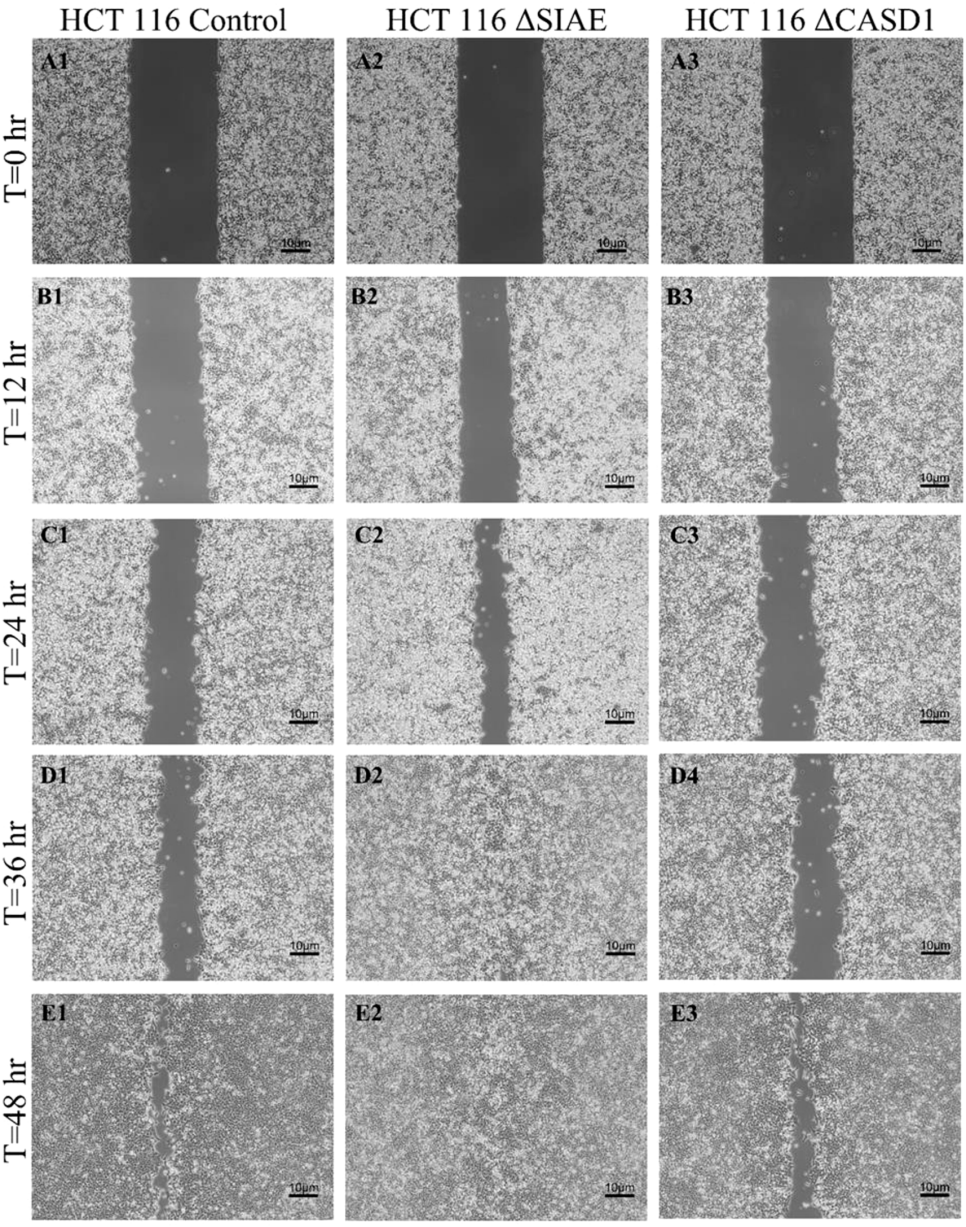
Scratch Migration Assay in HCT116 Cells. HCT 116 control (left), HCT 116 ΔSIAE (middle) and HCT 116 ΔCASD1 (right) cells were plated in a 6-well plate IBIDI insert with a 500 µm divider. The divider was carefully removed 12 hours after plating to create a uniform scratch. Cell migration into the scratch area was monitored by imaging every 12 hours over a period of 48 hours. Quantitative analysis of cell migration is presented in **Figure 14**.

**Figure 12:**
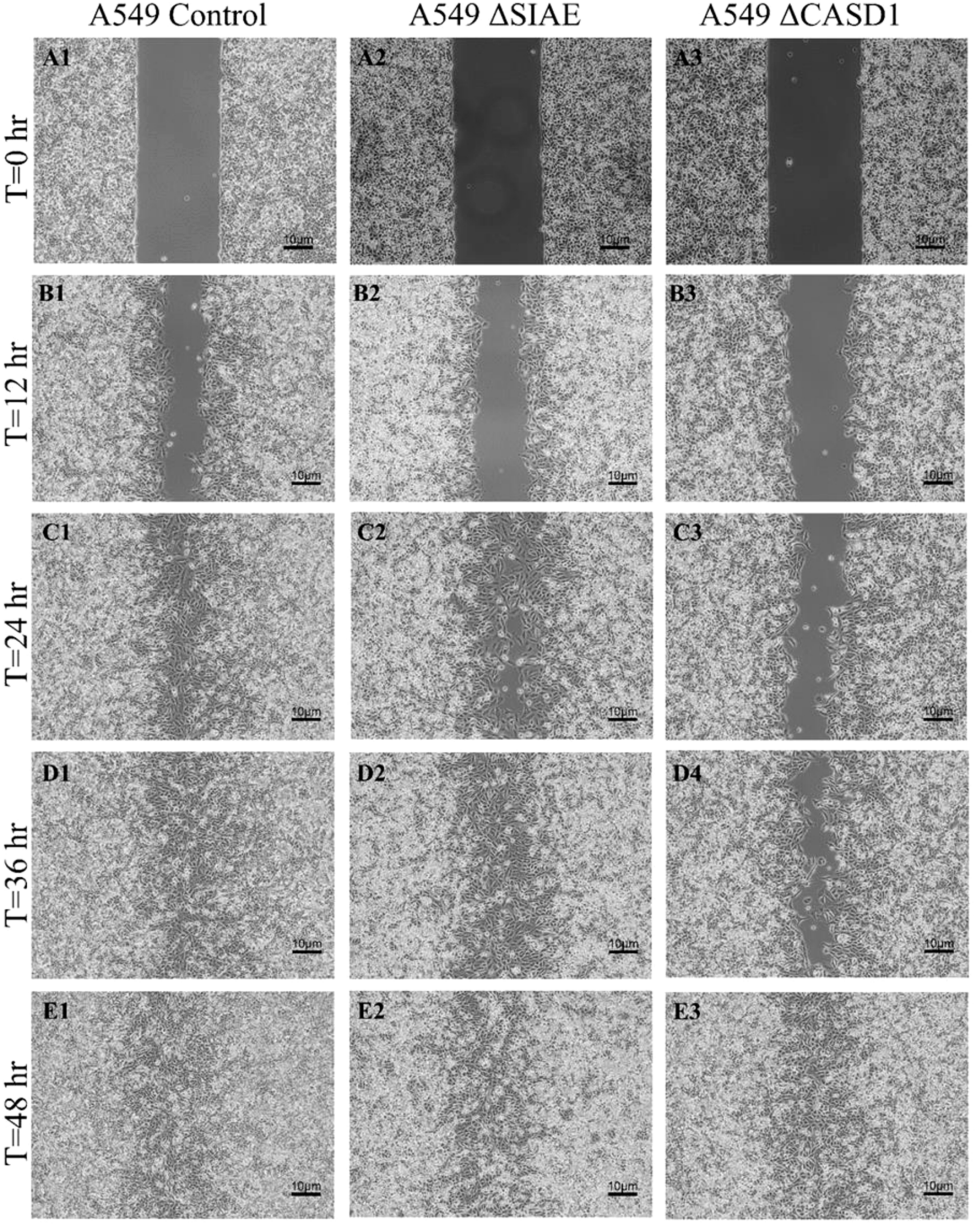
Scratch Migration Assay in A549 Cells. A549 control (left), A549 ΔSIAE (middle) and A549 ΔCASD1 (right) cells were plated in a 6-well plate IBIDI insert with a 500 µm divider. The divider was carefully removed 12 hours after plating to create a uniform scratch. Cell migration into the scratch area was monitored by imaging every 12 hours over a period of 48 hours. Quantitative analysis of cell migration is presented in **Figure 14**.

**Figure 13:**
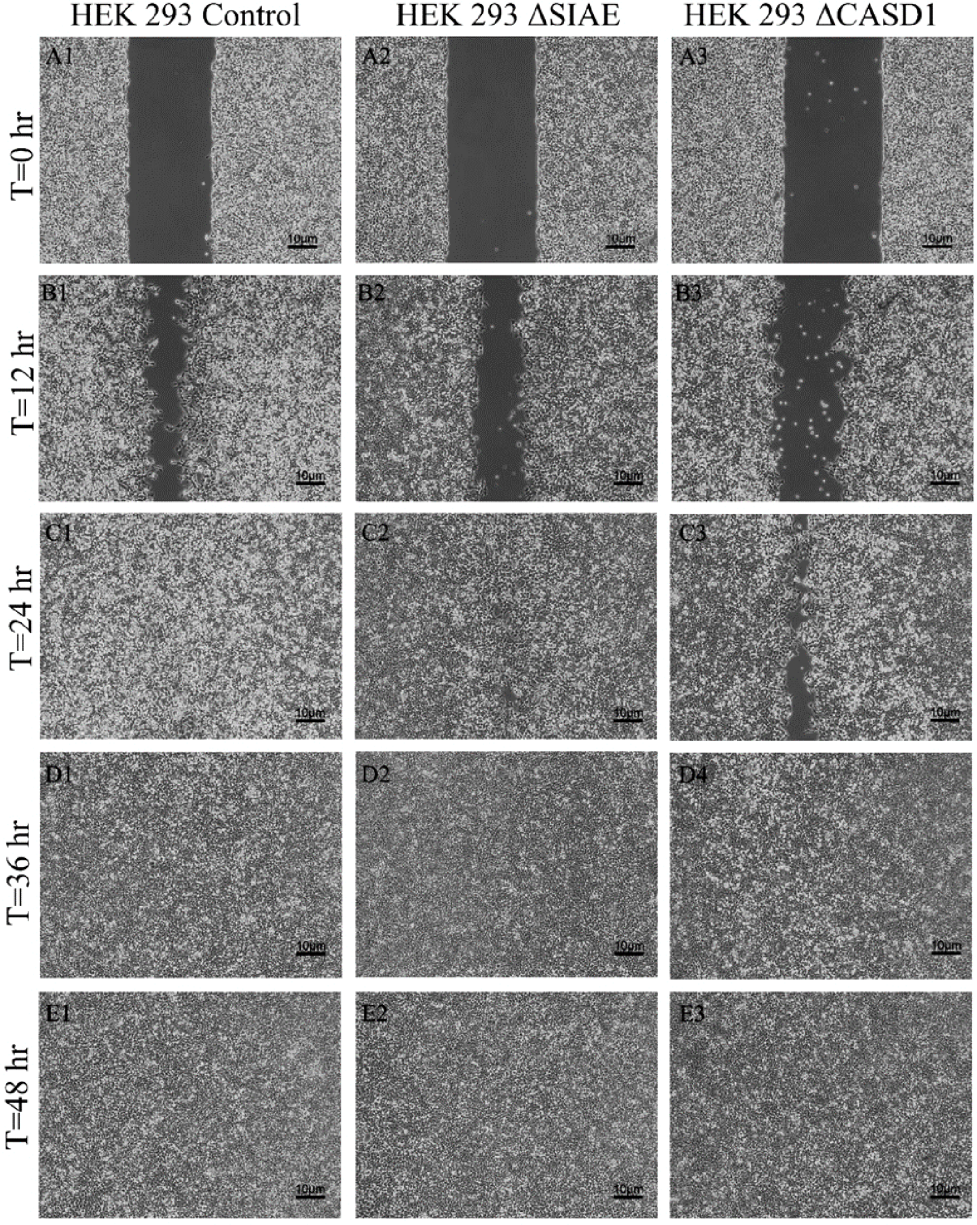
Scratch Migration Assay in HEK 293 Cells. HEK 293 control (left), HEK 293 ΔSIAE (middle) and HEK 293 ΔCASD1 (right) cells were plated in a 6-well plate IBIDI insert with a 500 µm divider. The divider was carefully removed 12 hours after plating to create a uniform scratch. Cell migration into the scratch area was monitored by imaging every 12 hours over a period of 48 hours. Quantitative analysis of cell migration is presented in.

**Figure 14:**
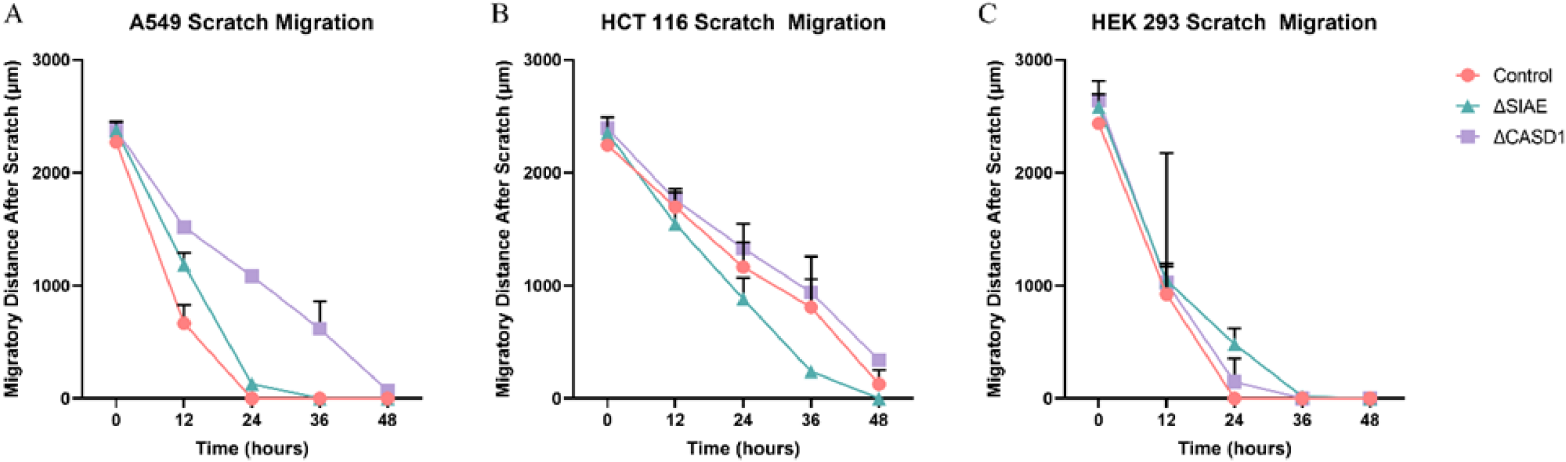
Scratch Assay Data. Migration measurements were analyzed using Fiji software for HCT 116 **(A)**, A549 **(B)**, and HEK 293 **(C)** cells. Data represent migration distances in micrometer.

After observing the effect of de-O-acetylated Sia on proliferation and chemotaxis of cancer cell lines, we sought to determine how modulating Sia contributes to the ability of cells to participate in cell-to-cell communication and migration. To this end, we performed a wound healing scratch assay. The cells’ ability to “close the gap,” migrate, appeared reduced when de-O-acetylated Sias were expressed on the cells’ surface, A549 ΔCASD1 (**Figure 11**), HCT116 ΔCASD1 (**Figure 12**), and HEK293 ΔCASD1 (**Figure 13**). This trend appeared consistent across all three cell lines (**Figure 14**).

## Discussion

The prominence of metastasis in cancer is unequivocal, standing as the foremost contributor to global cancer-related mortality, with nearly 90% of cancer deaths attributed to this complex process (6). Despite considerable advances in cell biology research and the proposal of various mechanistic insights surrounding metastasis, glycan related biological mechanisms of action and specifically the contribution of Sialic acid, remains incomplete (42). Despite being under studied, the Sia- Selectin pathway has garnered attention in recent years in shaping the complex terrain of metastasis (43).

Selectin receptors play a pivotal role in the early stages of leukocyte extravasation during inflammation or lymphocyte homing, orchestrating the gradual tethering and rolling of leukocytes along the vascular endothelium (4, 44, 45). Capitalizing on a comparable mechanism, cancer cells, equipped with their Selectin ligands, employ a parallel strategy to migrate and establish secondary tumors in distant sites (25, 46, 47).

Another pivotal hallmark of cancer emerges with the excessive transfer of sialic acid onto terminal glycoproteins or glycolipids, leading to ‘hypersialylation’ on the surface of tumor cells. This phenomenon serves multifaceted roles in cancer progression, particularly in the context of metastasis (34). Modeling has shown that increased placement of the negatively charged sialic acid on the cancer cell surface combined with decreased degradation induces robust cell-cell electrostatic repulsion, culminating in mechanical compression that facilitates cell detachment from the primary site and subsequent migration (34). Notably, hypersialylation also plays a protective role, shielding cancer cells from physiological pH stress. The anabolism of sialic acid yields two net protons, providing an advantageous mechanism for neutralizing the hydroxyl product of the Fenton reaction in cancer tissues (48).

Since Gunnar Blix’s groundbreaking discovery of sialic acid (49), over 80 distinct naturally occurring types have been identified to date. The extensive diversity in sialic acid structures stems from modifications to the hydroxyl and amine groups of the sialic acid backbone (50, 51). Among these modifications is the addition of acetyl groups to the hydroxyl groups at C-7, C-8, and C-9 positions (19), with O-acetylation emerging as the most extensively clinically studied modification (52).

In this study, we investigated the modulatory impact O-acetylation-Sias had on Selectin ligand expression, Selectin binding, and migratory effects. We explored how these binding alterations could influence the migratory capabilities of lung cancer (A549), colon cancer (HCT 116), and non-cancerous HEK293 cells. Employing CRISPR Cas9 gene editing techniques, we targeted the CAS1 domain containing 1 (CASD1) gene and sialic acid acetylesterase (SIAE) gene, responsible for adding and removing acetyl groups to C-9 and/or C-7 positions of Sia, respectively (53, 54). The CASD1 knockout (ΔCASD1) cells represented cancer cells with deacetylated sialic acid, while the SIAE knockout (ΔSIAE) cells represented cancer cells with acetylated sialic acid.

We firstly examined the effect of de-O-acetylation of sialic acid on the expression of Selectin ligands, focusing specifically on P-Selectin glycoprotein ligand-1 (PSGL-1) and Sialyl Lewis X(sLeX). Notably, increased sLeX expression has been inversely associated with cancer prognosis in human colon carcinoma and human non-small cell lung carcinoma [(41). Cancer cells employ various strategies to mimic monocytes or neutrophils, including the upregulation of sialylated Lewis-type blood group antigens (55). Although sLeX expression was traditionally attributed to internal glycosylation, recent discoveries have indicated that extrinsic glycosylation via platelets can also generate sLeX (56). PSGL-1 has been identified in multiple cell types, including most leukocytes and various cancer cells, such as human colon cancer, lymphomas, and prostate cancer (57–61). The results unequivocally demonstrated a substantial increase in the cell surface expression of both PSGL-1 (**Figure 2**) and sLeX (**Figure 3**) upon the induction of 9-O deacetylation of sialic acid. Because of the requirement for interaction between PSGL-1 on cancer cells and Selectin binding on the vascular endothelium after entry of the cancer cells into the bloodstream, this result holds implications for tethering and rolling processes in lung and colon cancers, shedding light on how increased PSGL-1 expression may impact the interactions between cancer cells and their microenvironment.

In the context of our investigation, previous studies have provided crucial insights into the underlying mechanisms influencing our findings. Notably, these studies have meticulously elucidated the pivotal role of sialic acid de-O-acetylation in unmasking Selectin ligands on the cancer cell surface. This unmasking phenomenon effectively renders the Selectin ligands more exposed than those of acetylated sialic acid and more accessible (2, 19).

We subsequently validated the modulatory role of deacetylated sialic acid by measuring the binding of E, P, and L Selectin-FITC conjugates to the cell surface. Recent studies have shed light on the role of Selectins in cancer. For instance, studies indicate that glioblastoma overexpresses P-Selectin in the presence of microglia and that P-Selectin plays a role in glioblastoma proliferation and invasion when microglia are present (62). Additionally, another study reported that in HT29 colon carcinoma cells, the interaction between E-Selectin and colon cancer cells has been linked to the activation of the PI3K/Akt pathway, suggesting a beneficial effect on cell survival (62). The cancer cell lines featuring CASD1 knockouts, indicative of deacetylated sialic acid, exhibited increased binding with all three Selectins (**Figures 5**–**6**). We conducted additional experiments on control cancer cell lines in which were treated with esterase probes to enzymatically cleave the acetyl groups. As expected, this treatment resulted in a significant increase in binding of both P- and L-Selectin, providing further confirmation of the crucial role of sialic acid de-O-acetylation in exposing Selectin ligands (**Figure 7**). These particular findings represent a novel contribution to the field, as previous research primarily focused on sLeX or sLeA but did not discuss the effect of functional group modification of sialic acid on sialic acid- Selectin binding (41, 63–65).

After elucidating the role of de-O-acetylated-Sia in binding to Selectins, our focus shifted to understanding how this binding could influence the migratory abilities of cancer cells. Subsequent assays were designed to elucidate the downstream effects of Selectin binding in the context of cancer metastasis. Our MTT assay data revealed the aggressive and proliferative characteristics associated with CASD1 knockout cancer cells compared to SIAE knockouts (**Figure 8**), aligning with findings from a separate study (12–14). The transwell assay did not show that exposure of cells to P and L selectin increased their migration. There was no effect above the 10% FBS control with the addition of Selectins. However, the transwell migration assay revealed that the migration of cells containing de-O-acetylated-Sia (CASD1 knockout cell lines) in CASD1 mutant cells in A549 cancer cells exceeded that of the control cell line but not in HCT116 (**Figures 9**, **10**). This may be attributed to the presence of low levels of 9-O-acetyled sialic acid internally as well as on the cell surface in control HCT116 cells, as demonstrated by our previous work (12). This could account for the comparable migration between HCT116 control cells and CASD1 knockout cells.

Our results suggest that soluble Selectins are not a stimulus for chemoattraction, as would be expected for an adhesion-based migration receptor-ligand interaction such as that between sialic acid and Selectins. These findings underscore the crucial role of de-O-acetylated sialic acid in influencing cell migration. Transwell migration assays are often used together with proliferation and scratch migration assays to determine the relative contributions of proliferation and migration, both important cellular features for the process of tumor progression (66). Upon analysis, our scratch assay data demonstrated that the migration ability of cells with de-O-acetylated sialic acid was lower compared to those with acetylated sialic acid (**Figures 11**–**14**). This finding underscores the specificity of Selectin’s impact on the migratory behavior observed in our study, distinct from any potential effects related to cellular proliferation. Furthermore, previous studies proposed that only epithelial cells distant from the initial wound show an increased rate of proliferation during the wound healing process, whereas cells migrating to cover the wound do not proliferate (67–70). Subsequent study suggested that the wound healing process, which assesses cell migration, is linked to characteristics associated with cellular mobility rather than cellular proliferation (66).

In conclusion, our study systematically unravels the impact of 9-O deacetylation of sialic acid on Selectin binding and cancer cell behavior. We observed a substantial increase in the cell surface expression of Selectin ligands upon 9-O deacetylation. This increase can be attributed, at least in part, to the role of sialic acid de-O-acetylation in exposing Selectin ligands on the surface of cancer cells. This result was further validated using Selectin-FITC conjugates, confirming increased binding with all three Selectin in CASD1 knockout cells. Additional experiments on SIAE knockout cell lines treated with esterase probes affirmed the pivotal role of sialic acid de-O-acetylation in Selectin binding. Our functional assays demonstrated heightened migration in CASD1 knockout cells, distinguishing the specific impact of sialic acid de-O-acetylation on migration from proliferation effects. The complex interplay between sialic acid and cancer cell behavior presents exciting opportunities for the development of targeted therapies and interventions that could significantly impact the field of oncology. While these results represent early *in vitro* studies, the results delineate for the first time the mechanistic contribution of de-O-acetylated-Sia to Selectin binding, reinforcing the importance of elucidating functional group alterations on Sia and their contribution. Without accurate identification of which functionalized form of Sia is being utilized to bind to sialic acid binding proteins,` Selectins for example, we cannot accurately produce glycan therapeutics with the correct specificity and reactivity as alternative treatment options. Thus, this work contributes integral foundational knowledge towards the development of promising glycan therapeutics in the realm of enzyme antibody drug conjugates.

## Data Availability

The original contributions presented in the study are included in the article/supplementary material. Further inquiries can be directed to the corresponding author.

## References

[1] Pinho SS Reis CA. Glycosylation in cancer: mechanisms and clinical implications. Nat Rev Cancer. (2015) 15:540–55. doi: 10.1038/nrc3982 26289314

[2] Mann B Klussmann E Vandamme-Feldhaus V Iwersen M Hanski ML Riecken EO. Low O-acetylation of sialyl-Le(x) contributes to its overexpression in colon carcinoma metastases. Int J Cancer. (1997) 72:258–64. doi: 10.1002/(SICI)1097-0215(19970717)72:2<258::AID-IJC10>3.0.CO;2-C 9219830

[3] Borsig L. Selectins in cancer immunity. Glycobiology. (2018) 28:648–55. doi: 10.1093/glycob/cwx105 PMC6711759 29272415

[4] Das K. Elucidating the role of sialic acid in tumorigenic pathways. In: Chemistry, Biochemistry and Physics. Brookings, South Dakota 57007: South Dakota State University (2024).

[5] Sahai E. Illuminating the metastatic process. Nat Rev Cancer. (2007) 7:737–49. doi: 10.1038/nrc2229 17891189

[6] Seyfried TN Huysentruyt LC. On the origin of cancer metastasis. Crit Rev Oncog. (2013) 18:43–73. doi: 10.1615/CritRevOncog.v18.i1-2.40 23237552 PMC3597235

[7] Stowell SR Ju T Cummings RD. Protein glycosylation in cancer. Annu Rev Pathol. (2015) 10:473–510. doi: 10.1146/annurev-pathol-012414-040438 25621663 PMC4396820

[8] Bull C Stoel MA Brok den MH Adema GJ. Sialic acids sweeten a tumor’s life. Cancer Res. (2014) 74:3199–204. doi: 10.1158/0008-5472.CAN-14-0728 24830719

[9] Scupakova K Adelaja OT Balluff B Ayyappan V Tressler CM Jenkinson NM. Clinical importance of high-mannose, fucosylated, and complex N-glycans in breast cancer metastasis. JCI Insight. (2021) 6. doi: 10.1172/jci.insight.146945 PMC8783675 34752419

[10] Barkeer S Chugh S Batra SK Ponnusamy MP. Glycosylation of cancer stem cells: function in stemness, tumorigenesis, and metastasis. Neoplasia. (2018) 20:813–25. doi: 10.1016/j.neo.2018.06.001 PMC6037882 30015157

[11] Tvaroska I Selvaraj C Koca J. Selectins-the two Dr. Jekyll and Mr. Hyde faces of adhesion molecules-A review. Molecules. (2020) 25. doi: 10.3390/molecules25122835 PMC7355470 32575485

[12] Grabenstein S Barnard KN Anim M Armoo A Weichert WS Bertozzi CR. Deacetylated sialic acids modulates immune mediated cytotoxicity via the sialic acid-Siglec pathway. Glycobiology. (2021) 31:1279–94. doi: 10.1093/glycob/cwab068 34192335

[13] Tuffour I Amuzu S Bayoumi H Surtaj I Parrish C Willand-Charnley R. Early in vitro evidence indicates that deacetylated sialic acids modulate multi-drug resistance in colon and lung cancers via breast cancer resistance protein. Front Oncol. (2023) 13:1145333. doi: 10.3389/fonc.2023.1145333 37377914 PMC10291187

[14] Tuffour I. Modulatory effects of deacetylated sialic acids on breast cancer resistance protein-mediated multidrug resistance and receptor tyrosine kinase-targeted therapy. In: Chemistry, Biochemistry, and Physics. Brookings, South Dakota: South Dakota State Univeersity (2023).

[15] Wilson DF Massey W. Scanning electron microscopy of incinerated teeth. Am J Forensic Med Pathol. (1987) 8:32–8. doi: 10.1097/00000433-198703000-00008 3578204

[16] Lise M Belluco C Perera SP Patel R Thomas P Ganguly A. Clinical correlations of alpha2,6-sialyltransferase expression in colorectal cancer patients. Hybridoma. (2000) 19:281–6. doi: 10.1089/027245700429828 11001400

[17] Sata T Roth J Zuber C Stamm B Heitz P. Expression of alpha 2,6-linked sialic acid residues in neoplastic but not in normal human colonic mucosa. A lectin-gold cytochemical study with Sambucus nigra and Maackia amurensis lectins. Am J Pathol. (1991) 139:1435–48. PMC1886452 1661075

[18] van Kooyk Y Rabinovich GA. Protein-glycan interactions in the control of innate and adaptive immune responses. Nat Immunol. (2008) 9:593–601. doi: 10.1038/ni.f.203 18490910

[19] Visser EA Moons SJ Timmermans SBPE de Jong H Boltje TJ Büll C. Sialic acid O-acetylation: From biosynthesis to roles in health and disease. J Biol Chem. (2021) 297:100906. doi: 10.1016/j.jbc.2021.100906 34157283 PMC8319020

[20] Amon R Reuven EM Leviatan Ben-Arye S Padler-Karavani V. Glycans in immune recognition and response. Carbohydr Res. (2014) 389:115–22. doi: 10.1016/j.carres.2014.02.004 24680512

[21] McEver RP. Selectin-carbohydrate interactions during inflammation and metastasis. Glycoconj J. (1997) 14:585–91. doi: 10.1023/A:1018584425879 9298691

[22] Munkley J Scott E. Targeting aberrant sialylation to treat cancer. Medicines (Basel). (2019) 6. doi: 10.3390/medicines6040102 PMC6963943 31614918

[23] Smith BAH Bertozzi CR. The clinical impact of glycobiology: targeting selectins, Siglecs and mammalian glycans. Nat Rev Drug Discovery. (2021) 20:217–43. doi: 10.1038/s41573-020-00093-1 PMC7812346 33462432

[24] Mahoney TS Weyrich AS Dixon DA McIntyre T Prescott SM Zimmerman GA. Cell adhesion regulates gene expression at translational checkpoints in human myeloid leukocytes. Proc Natl Acad Sci U.S.A. (2001) 98:10284–9. doi: 10.1073/pnas.181201398 PMC56953 11517314

[25] Natoni A Macauley MS O’Dwyer ME. Targeting selectins and their ligands in cancer. Front Oncol. (2016) 6:93. doi: 10.3389/fonc.2016.00093 27148485 PMC4834419

[26] McEver RP. Selectins: lectins that initiate cell adhesion under flow. Curr Opin Cell Biol. (2002) 14:581–6. doi: 10.1016/S0955-0674(02)00367-8 12231353

[27] Ley K. The role of selectins in inflammation and disease. Trends Mol Med. (2003) 9:263–8. doi: 10.1016/S1471-4914(03)00071-6 12829015

[28] Pearce OM Laubli H. Sialic acids in cancer biology and immunity. Glycobiology. (2016) 26:111–28. doi: 10.1093/glycob/cwv097 26518624

[29] Kansas GS. Selectins and their ligands: current concepts and controversies. Blood. (1996) 88:3259–87. doi: 10.1182/blood.V88.9.3259.bloodjournal8893259 8896391

[30] Borsig L Vlodavsky I Ishai-Michaeli R Torri G Vismara E. Sulfated hexasaccharides attenuate metastasis by inhibition of P-selectin and heparanase. Neoplasia. (2011) 13:445–52. doi: 10.1593/neo.101734 PMC3084621 21532885

[31] Zak I Lewandowska E Gnyp W. Selectin glycoprotein ligands. Acta Biochim Pol. (2000) 47:393–412. doi: 10.18388/abp.2000_4019 11051204

[32] Zhao S Wang Z. Changes in heparan sulfate sulfotransferases and cell-surface heparan sulfate during SKM-1 cells granulocytic differentiation and A549 cells epithelial-mesenchymal transition. Glycoconj J. (2020) 37:151–64. doi: 10.1007/s10719-019-09903-0 31863309

[33] McEver RP. Selectins: initiators of leucocyte adhesion and signalling at the vascular wall. Cardiovasc Res. (2015) 107:331–9. doi: 10.1093/cvr/cvv154 PMC4592324 25994174

[34] Esposito M Mondal N Greco TM Wei Y Spadazzi C Lin SC. Bone vascular niche E-selectin induces mesenchymal-epithelial transition and Wnt activation in cancer cells to promote bone metastasis. Nat Cell Biol. (2019) 21:627–39. doi: 10.1038/s41556-019-0309-2 PMC6556210 30988423

[35] Hauselmann I Roblek M Protsyuk D Huck V Knopfova L Grässle S. Monocyte induction of E-selectin-mediated endothelial activation releases VE-cadherin junctions to promote tumor cell extravasation in the metastasis cascade. Cancer Res. (2016) 76:5302–12. doi: 10.1158/0008-5472.CAN-16-0784 PMC6322653 27488527

[36] Huang J Huang J Zhang G. Insights into the role of sialylation in cancer metastasis, immunity, and therapeutic opportunity. Cancers (Basel). (2022) 14. doi: 10.3390/cancers14235840 PMC9737300 36497322

[37] Smith BAH Bertozzi CR. Author Correction: The clinical impact of glycobiology: targeting selectins, Siglecs and mammalian glycans. Nat Rev Drug Discovery. (2021) 20:244. doi: 10.1038/s41573-021-00160-1 33558696 PMC8095272

[38] Kappelmayer J Nagy B Jr. The interaction of selectins and PSGL-1 as a key component in thrombus formation and cancer progression. BioMed Res Int. (2017) 2017:6138145. doi: 10.1155/2017/6138145 28680883 PMC5478826

[39] Hoos A Protsyuk D Borsig L. Metastatic growth progression caused by PSGL-1-mediated recruitment of monocytes to metastatic sites. Cancer Res. (2014) 74:695–704. doi: 10.1158/0008-5472.CAN-13-0946 24322980

[40] ChaChadi VB Bhat G Cheng PW. Glycosyltransferases involved in the synthesis of MUC-associated metastasis-promoting selectin ligands. Glycobiology. (2015) 25:963–75. doi: 10.1093/glycob/cwv030 PMC4518684 25972125

[41] St Hill CA Baharo-Hassan D Farooqui M. C2-O-sLeX glycoproteins are E-selectin ligands that regulate invasion of human colon and hepatic carcinoma cells. PloS One. (2011) 6:e16281. doi: 10.1371/journal.pone.0016281 21283832 PMC3023807

[42] Fares J Fares MY Khachfe HH Salhab HA Fares Y. Molecular principles of metastasis: a hallmark of cancer revisited. Signal Transduct Target Ther. (2020) 5:28. doi: 10.1038/s41392-020-0134-x 32296047 PMC7067809

[43] Lange T Valentiner U Wicklein D Maar H Labitzky V Ahlers AK. Tumor cell E-selectin ligands determine partialefficacy of bortezomib on spontaneous lung metastasis formation of solid human tumors in vivo. Mol Ther. (2022) 30:1536–52. doi: 10.1016/j.ymthe.2022.01.017 PMC9077315 35031433

[44] Steeber DA Campbell MA Basit A Ley K Tedder TF. Optimal selectin-mediated rolling of leukocytes during inflammation in vivo requires intercellular adhesion molecule-1 expression. Proc Natl Acad Sci U.S.A. (1998) 95:7562–7. doi: 10.1073/pnas.95.13.7562 PMC22683 9636189

[45] Norman KE Moore KL McEver RP Ley K. Leukocyte rolling in vivo is mediated by P-selectin glycoprotein ligand-1. Blood. (1995) 86:4417–21. doi: 10.1182/blood.V86.12.4417.bloodjournal86124417 8541529

[46] Gassmann P Enns A Haier J. Role of tumor cell adhesion and migration in organ-specific metastasis formation. Onkologie. (2004) 27:577–82. doi: 10.1159/000081343 15591720

[47] Gout S Tremblay PL Huot J. Selectins and selectin ligands in extravasation of cancer cells and organ selectivity of metastasis. Clin Exp Metastasis. (2008) 25:335–44. doi: 10.1007/s10585-007-9096-4 17891461

[48] Sun H Zhou Y Jiang H Xu. Elucidation of functional roles of sialic acids in cancer migration. Front Oncol. (2020) 10:401. doi: 10.3389/fonc.2020.00401 32296639 PMC7137995

[49] Lundblad A. Gunnar Blix and his discovery of sialic acids. Fascinating molecules in glycobiology. Ups J Med Sci. (2015) 120:104–12. doi: 10.3109/03009734.2015.1027429 PMC4463483 25921326

[50] Tyagi W Pandey V Pokharel YR. Membrane linked RNA glycosylation as new trend to envision epi-transcriptome epoch. Cancer Gene Ther. (2023) 30:641–6. doi: 10.1038/s41417-022-00430-z 35136215

[51] Schauer R. Chapter One - Exploration of the Sialic Acid World, in Advances in Carbohydrate Chemistry and Biochemistry. Baker DC, editor. Academic Press (2018) p. 1–213. 2009: Cold Spring Harbor Press. 10.1016/bs.accb.2018.09.001 PMC7112061 30509400

[52] Varki A Cummings RD Esko JD Stanley P Hart GW Aebi M. Essentials of glycobiology. 4th edition. (2009).

[53] Janbon G Himmelreich U Moyrand F Improvisi L Dromer F. Cas1p is a membrane protein necessary for the O-acetylation of the Cryptococcus neoformans capsular polysaccharide. Mol Microbiol. (2001) 42:453–67. doi: 10.1046/j.1365-2958.2001.02651.x 11703667

[54] Takematsu H Diaz S Stoddart A Zhang Y Varki A. Lysosomal and cytosolic sialic acid 9-O-acetylesterase activities can Be encoded by one gene via differential usage of a signal peptide-encoding exon at the N terminus. J Biol Chem. (1999) 274:25623–31. doi: 10.1074/jbc.274.36.25623 10464298

[55] Gomes C. Expression of ST3GAL4 leads to SLe(x) expression and induces c-Met activation and an invasive phenotype in gastric carcinoma cells. PloS One. (2013) 8:e66737. doi: 10.1371/journal.pone.0066737 23799130 PMC3682978

[56] Reily C Stewart TJ Renfrow MB Novak J. Glycosylation in health and disease. Nat Rev Nephrol. (2019) 15:346–66. doi: 10.1038/s41581-019-0129-4 PMC6590709 30858582

[57] Pereira JL Cavaco P da Silva RC Pacheco-Leyva I Mereiter S Pinto R. P-selectin glycoprotein ligand 1 promotes T cell lymphoma development and dissemination. Transl Oncol. (2021) 14:101125. doi: 10.1016/j.tranon.2021.101125 34090013 PMC8188565

[58] Dimitroff CJ Descheny L Trujillo N Kim R Nguyen V Huang W. Identification of leukocyte E-selectin ligands, P-selectin glycoprotein ligand-1 and E-selectin ligand-1, on human metastatic prostate tumor cells. Cancer Res. (2005) 65:5750–60. doi: 10.1158/0008-5472.CAN-04-4653 PMC1472661 15994950

[59] Kauffman K Manfra D Nowakowska D Zafari M Nguyen PA Phennicie R. PSGL-1 blockade induces classical activation of human tumor-associated macrophages. Cancer Res Commun. (2023) 3:2182–94. doi: 10.1158/2767-9764.CRC-22-0513 PMC10601817 37819238

[60] Izumi Y Kawamura YJ Irimura T. Carbohydrate antigens in carcinoma invasion and metastasis. Nihon Geka Gakkai Zasshi. (1996) 97:140–4. 8632742

[61] Okamura N Mori Y Endo T Ito E Kudo R. Experimental studies on the cell adhesion molecule E-cadherin and in vitro invasion of endometrial carcinoma cell lines. Nihon Sanka Fujinka Gakkai Zasshi. (1996) 48:335–42. 8847459

[62] Yeini E Ofek P Pozzi S Albeck N Ben-Shushan D Tiram G. P-selectin axis plays a key role in microglia immunophenotype and glioblastoma progression. Nat Commun. (2021) 12:1912. doi: 10.1038/s41467-021-22186-0 33771989 PMC7997963

[63] Trinchera M Aronica A Dall’Olio F. Selectin ligands sialyl-lewis a and sialyl-lewis x in gastrointestinal cancers. Biol (Basel). (2017) 6. doi: 10.3390/biology6010016 PMC5372009 28241499

[64] Chen SH Dallas MR Balzer EM Konstantopoulos. Mucin 16 is a functional selectin ligand on pancreatic cancer cells. FASEB J. (2012) 26:1349–59. doi: 10.1096/fj.11-195669 PMC3289508 22159147

[65] Sakuma K Aoki M Kannagi R. Transcription factors c-Myc and CDX2 mediate E-selectin ligand expression in colon cancer cells undergoing EGF/bFGF-induced epithelial-mesenchymal transition. Proc Natl Acad Sci U.S.A. (2012) 109:7776–81. doi: 10.1073/pnas.1111135109 PMC3356678 22547830

[66] Flate E Stalvey JR. Motility of select ovarian cancer cell lines: effect of extra-cellular matrix proteins and the involvement of PAK2. Int J Oncol. (2014) 45:1401–11. doi: 10.3892/ijo.2014.2553 PMC4151804 25050916

[67] Guo X Hutcheon AE Zieske JD. TAT-mediated protein transduction into human corneal epithelial cells: p15(INK4b) inhibits cell proliferation and stimulates cell migration. Invest Ophthalmol Vis Sci. (2004) 45:1804–11. doi: 10.1167/iovs.03-1164 15161843

[68] Sharma GD He J Bazan HE. p38 and ERK1/2 coordinate cellular migration and proliferation in epithelial wound healing: evidence of cross-talk activation between MAP kinase cascades. J Biol Chem. (2003) 278:21989–97. doi: 10.1074/jbc.M302650200 12663671

[69] Zagon IS Sassani JW McLaughlin PJ. Cellular dynamics of corneal wound re-epithelialization in the rat. II. DNA synthesis of the ocular surface epithelium following wounding. Brain Res. (1999) 839:243–52. doi: 10.1016/S0006-8993(99)01722-9 10519047

[70] Chung EH Hutcheon AE Joyce NC Zieske JD. Synchronization of the G1/S transition in response to corneal debridement. Invest Ophthalmol Vis Sci. (1999) 40:1952–8. 10440248

